# Genuine and Sequestered Natural Products from the Genus *Orobanche* (Orobanchaceae, Lamiales)

**DOI:** 10.3390/molecules23112821

**Published:** 2018-10-30

**Authors:** Friederike Scharenberg, Christian Zidorn

**Affiliations:** Pharmazeutisches Institut, Abteilung Pharmazeutische Biologie, Christian-Albrechts-Universität zu Kiel, Gutenbergstraße 76, 24118 Kiel, Germany; fscharenberg@pharmazie.uni-kiel.de

**Keywords:** *Orobanche* s.l., Orobanchaceae, Lamiales, natural products, secondary metabolites, phenylpropanoid glycosides, phenylethanoid glycosides, bioactivities of natural products, chemosystematics

## Abstract

The present review gives an overview about natural products from the holoparasitic genus *Orobanche* (Orobanchaceae). We cover both genuine natural products as well as compounds sequestered by *Orobanche* taxa from their host plants. However, the distinction between these two categories is not always easy. In cases where the respective authors had not indicated the opposite, all compounds detected in *Orobanche* taxa were regarded as genuine *Orobanche* natural products. From the about 200 species of *Orobanche* s.l. (i.e., including *Phelipanche*) known worldwide, only 26 species have so far been investigated phytochemically (22 *Orobanche* and four *Phelipanche* species), from 17 *Orobanche* and three *Phelipanche* species defined natural products (and not only natural product classes) have been reported. For two species of *Orobanche* and one of *Phelipanche* dedicated studies have been performed to analyze the phenomenon of natural product sequestration by parasitic plants from their host plants. In total, 70 presumably genuine natural products and 19 sequestered natural products have been described from *Orobanche* s.l.; these form the basis of 140 chemosystematic records (natural product reports per taxon). Bioactivities described for *Orobanche* s.l. extracts and natural products isolated from *Orobanche* species include in addition to antioxidative and anti-inflammatory effects, e.g., analgesic, antifungal and antibacterial activities, inhibition of amyloid β aggregation, memory enhancing effects as well as anti-hypertensive effects, inhibition of blood platelet aggregation, and diuretic effects. Moreover, muscle relaxant and anti-spasmodic effects as well as anti-photoaging effects have been described.

## 1. Introduction

Taxa of the genus *Orobanche* sensu lato (Orobanchaceae) are non-photosynthetic root holoparasites. The genus *Orobanche* s.l. is distributed worldwide. Most of the about 200 species are native to the Northern Hemisphere [1,2]. The phylogeny of *Orobanche* s.l. is still discussed controversially. Park et al., (2008) [3], distinguished five lineages within *Orobanche* s.l. based on morphological, cytological, and (macro-)molecular phylogenetic data. Schneeweiss (2013) [4] suggested treating these lineages as separate genera. From these five lineages, four have traditionally been regarded as sections of *Orobanche* [the respective generic names suggested by Schneeweiss (2013) [4] are indicated in brackets]: *Gymnocaulis* Nutt. (=genus *Aphyllon* Mitch.), *Myzorrhiza* (Phil.) Beck (=sect. *Nothaphyllon* (A.Gray) Heckard = genus *Myzorrhiza* Phil.) [5], *Trionychon* Wallr. (=genus *Phelipanche* Pomel), and *Orobanche* L. (=sect. *Osproleon* Wallr. = genus *Orobanche* L. s.str.). The only representative of the fifth clade, *Orobanche latisquama* Reut. ex Boiss. (=genus *Boulardia* F.W.Schultz), was formerly assigned to sect. *Orobanche*. Additionally, the genus *Phelypaea* is part of the clade comprising *Orobanche* s.l. [4]. On the basis of recent taxonomic and phylogenetic data, the genus *Orobanche* s.l. was split into two separate genera, *Orobanche* and *Phelipanche* [6]. Following this decision the names *Phelipanche ramosa* (L.) Pomel and *P. aegyptiaca* (Pers.) Pomel, instead of *Orobanche ramosa* L. and *O. aegyptiaca* Pers. (of sect. *Trionychon*) are applicable [7]. In the following paragraphs, the new species names are used and synonyms in the original publications are indicated if applicable. *Orobanche* species play an important (negative) role in agriculture as they can pose serious threats to major crop plants [8,9]. Many research papers are focused on host-parasite interactions via strigolactones (seed germination promoters) in terms of weed management. Less is known about phytochemical compounds in the parasitic species. This review is intended to give an overview over secondary metabolites synthesized by *Orobanche* s.l. known so far. For *Orobanche* sections *Gymnocaulis* and *Myzorrhiza* as well as for *O. latisquama,* no studies on secondary metabolites have been published yet. Members of *Orobanche* section *Orobanche* and of *Phelipanche* that have been phytochemically analyzed are reviewed in alphabetical order. An additional paragraph describes sequestration of secondary metabolites from host plants. Characterizations of bioactivities of *Orobanche* s.l. extracts and pure compounds isolated from *Orobanche* species are described separately. Literature data were retrieved from SciFinder and ISI Web of Knowledge databases. Reports until the end of 2017 were taken into account (exact search terms can be found in Supplementary Text S1). All taxon names were reviewed and accepted names according to The Plant List are used throughout the manuscript [10]. Usage of synonyms deviating from The Plant List in the cited literature was mentioned when applicable.

## 2. Results and Discussion

### 2.1. Literature Data on Natural Products from Orobanche Species

**Figure 1:**
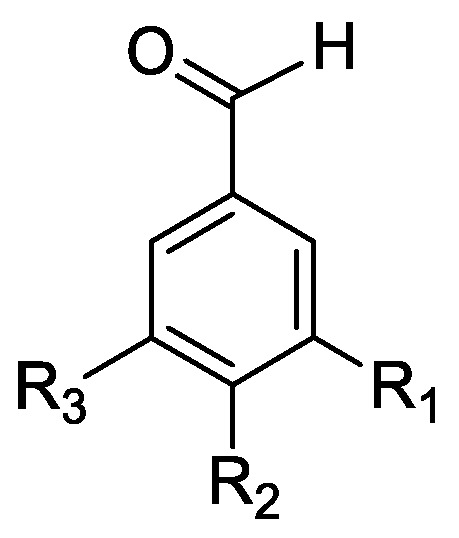
Phenylmethanoids, aromatic aldehydes and ketones **1**–**5**.

**Figure 2:**
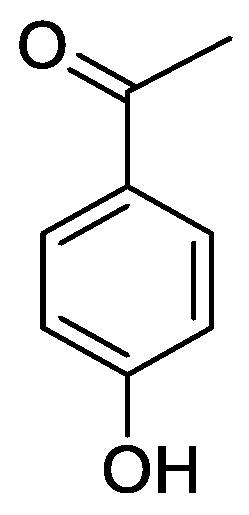
Phenylethanoid, *p*-Hydroxy acetophenone **6**.

**Figure 3:**
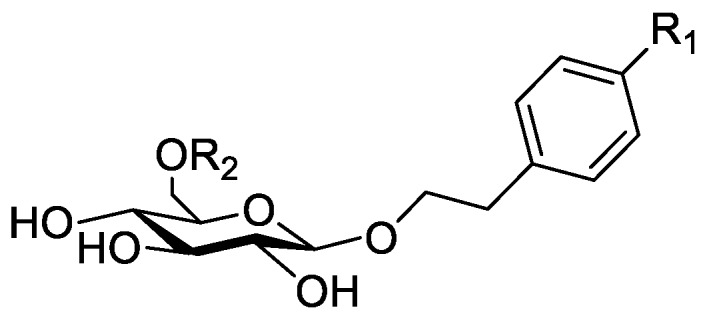
Non-acylated phenylethanoid glycosides **7** and **8**.

**Figure 4:**
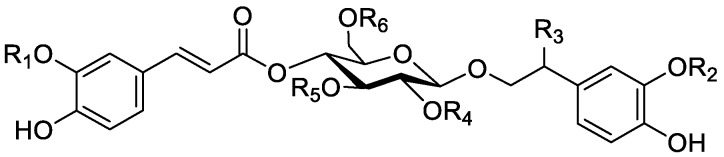
Phenylpropanoid glycosides I, **9**–**22**.

**Figure 5:**
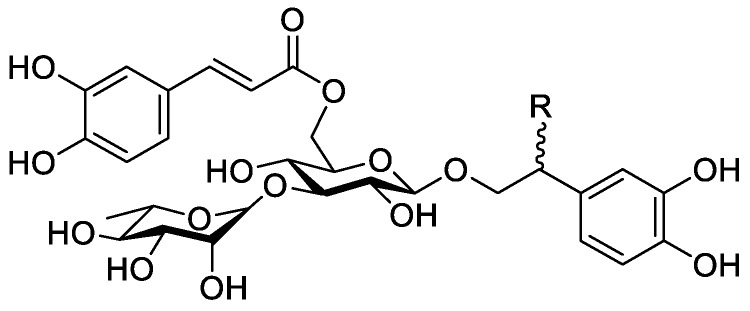
Phenylpropanoid glycosides II, **23** and **24**.

**Figure 6:**
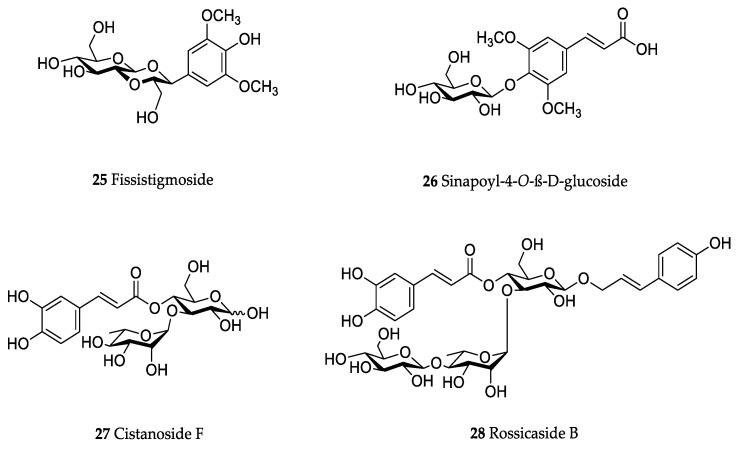
Phenylpropanoid glycosides III, **25**–**28**.

**Figure 7:**
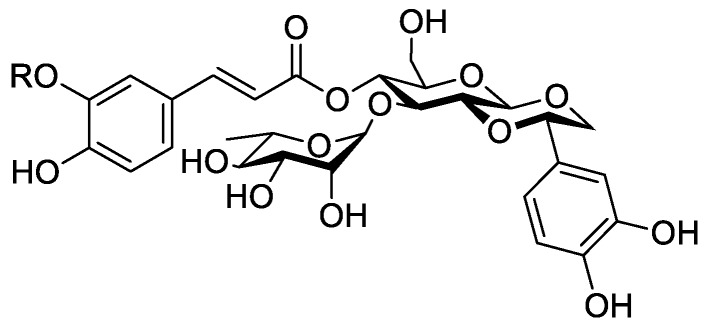
Phenylpropanoid glycosides IV, **29** and **30**. Note: According to Andary et al. [38] crenatoside and oraposide both describe the same structure. The authors report another isolated substance, orobanchoside (also mentioned in a former work from Andary et al. [39]). In a later work from Nishibe et al. [34] the structure of orobanchoside is being revised and it was established that orobanchoside is the same compound as crenatoside and oraposide. The name oraposide is used preferentially here, because it seems to be the oldest name for the compound at hand; however, crenatoside seems to have been used more often in the literature.

**Figure 8:**
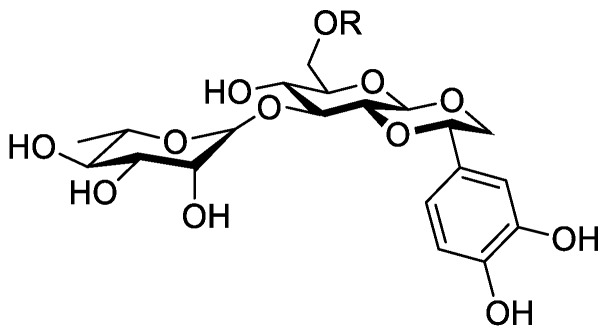
Phenylpropanoid glycosides V, **31** and **32**, and phenylethanoid glycoside **33**.

**Figure 9:**
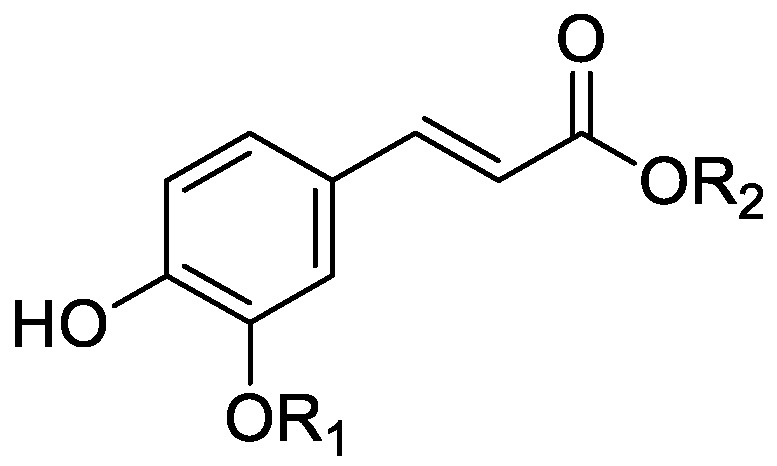
Phenolic acids **34**–**36**.

**Figure 10:**
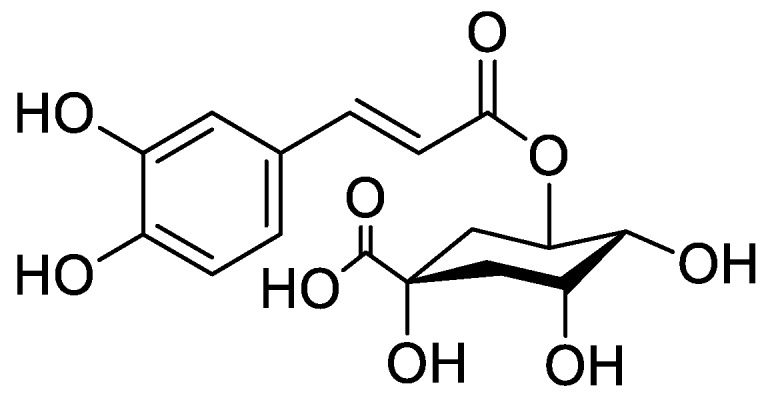
Chlorogenic acid (5-Caffeoyl quinic acid) **37**.

**Figure 11:**
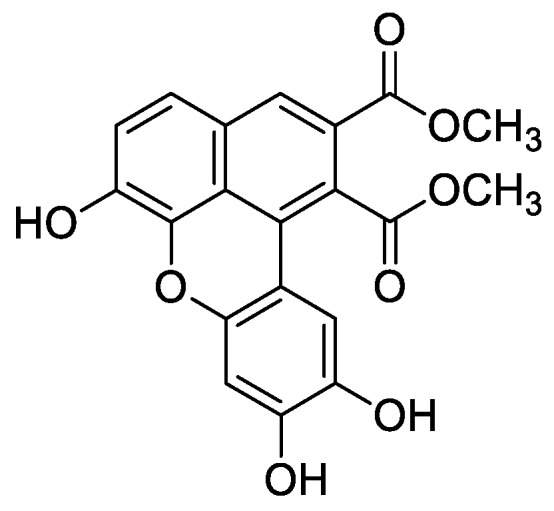
Dimethyl-6,9,10-trihydroxybenzol[*kl*]xanthene-1,2-dicarboxylate **38**.

**Figure 12:**
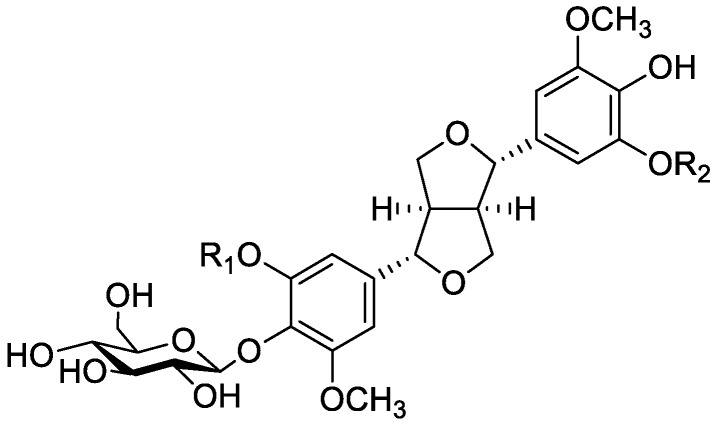
Lignans **39**–**41**.

**Figure 13:**
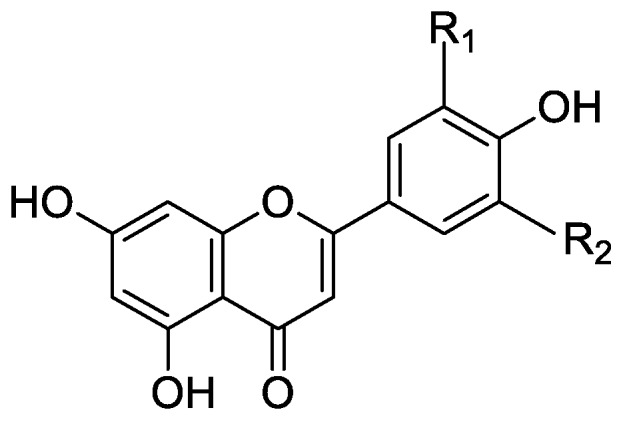
Flavones **42**–**44**.

**Figure 14:**
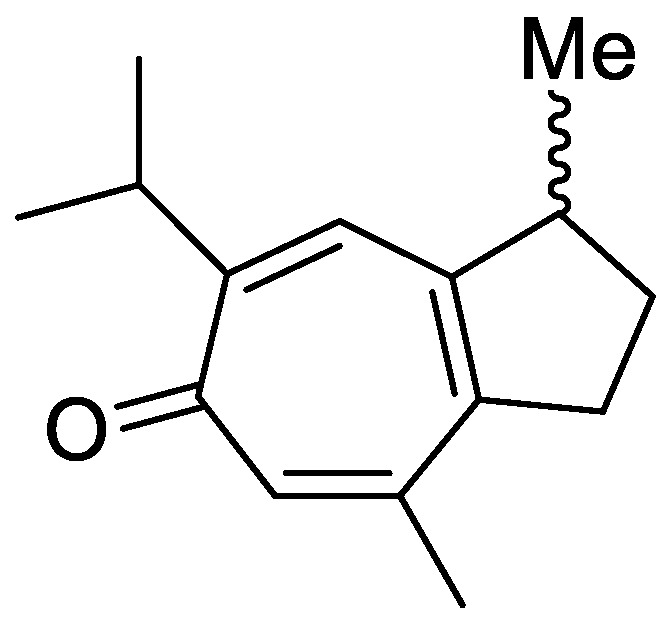
Tropone derivative—Orobanone **45**.

**Figure 15:**
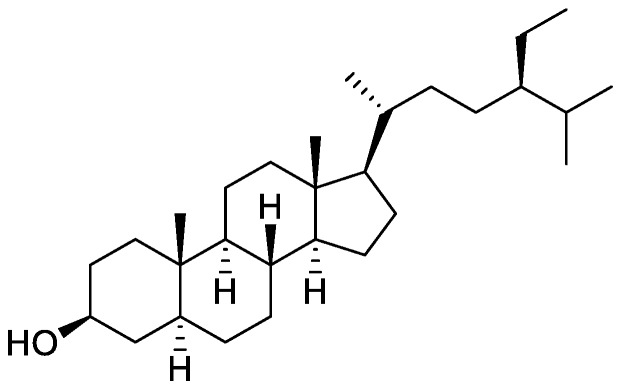
Sterols I: stigmastane derivative, stigmastanol **46**.

**Figure 16:**
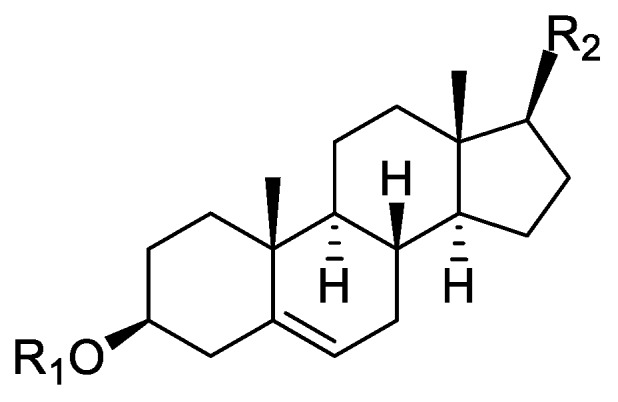
Sterols II: cholest-5-en derivatives, **47**–**54**.

**Figure 17:**
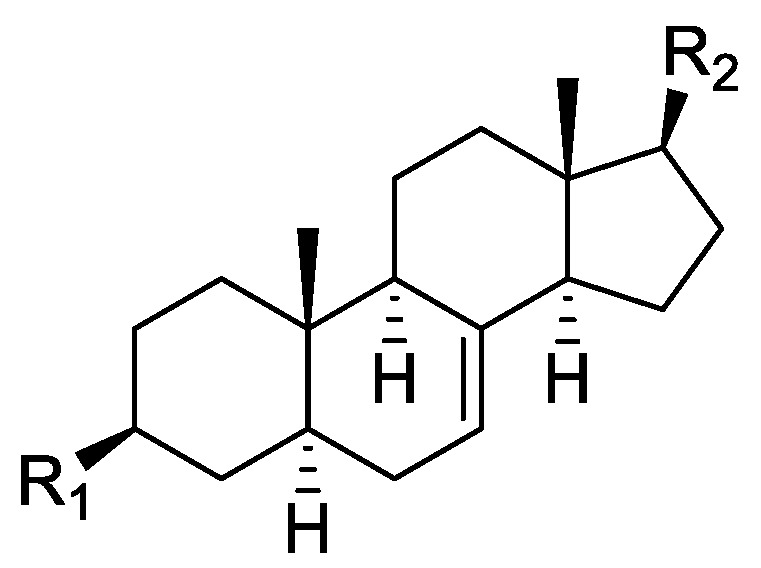
Sterols III: cholest-7-en derivatives, **55**–**58**.

**Figure 18:**
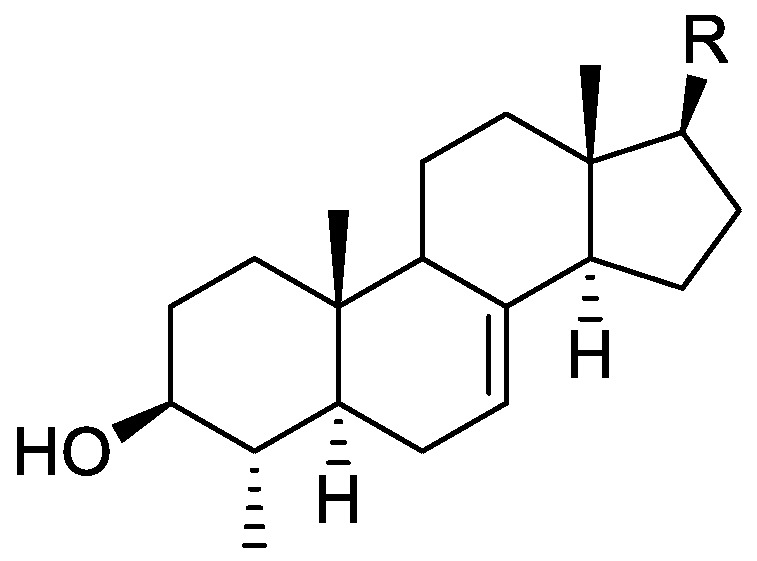
Sterols IV: lophenol derivatives, **59**–**62**.

**Figure 19:**
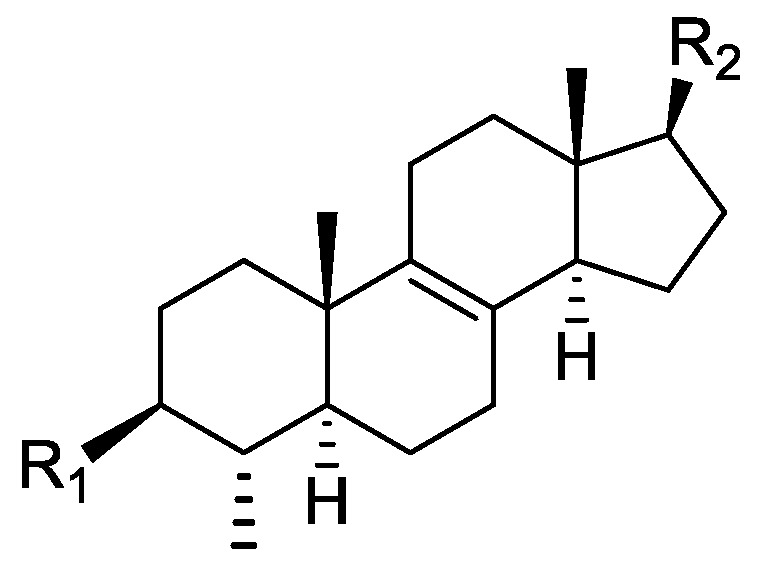
Sterols V, **63**–**66**.

**Figure 20:**
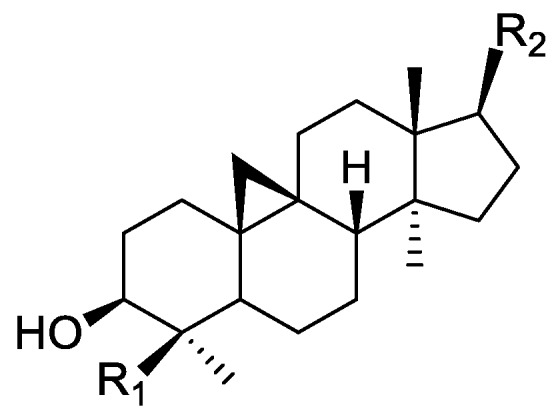
Sterols VI, **67**–**70**.

**Figure 21:**
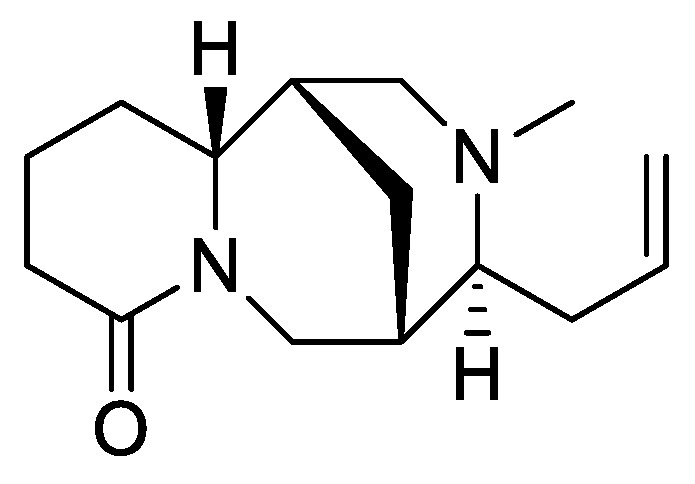
N-Methylangustifoline **S1**.

**Figure 22:**
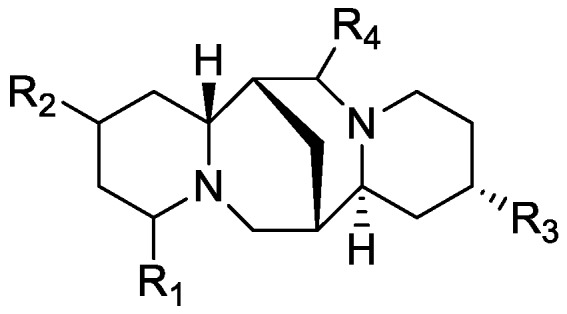
Quinolizidine alkaloids I, **S2**–**S10**.

**Figure 23:**
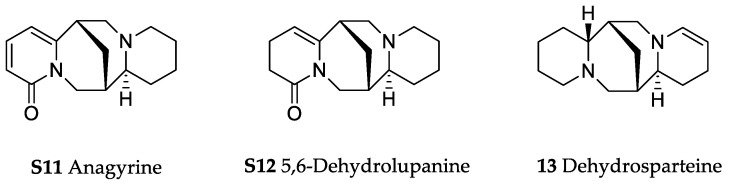
Quinolizidine alkaloids II, **S11**–**S13**.

**Figure 24:**
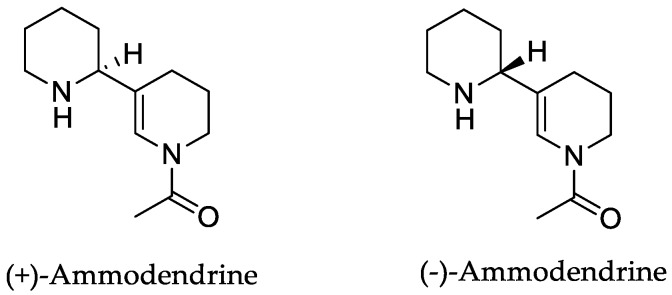
Piperidine alkaloid—Ammodendrine **S14**.

**Figure 25:**
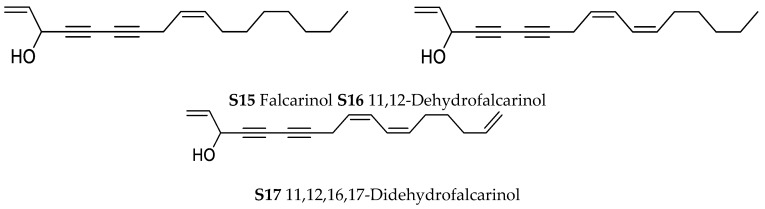
Polyacetylenes **S15**–**S17**.

**Figure 26:**
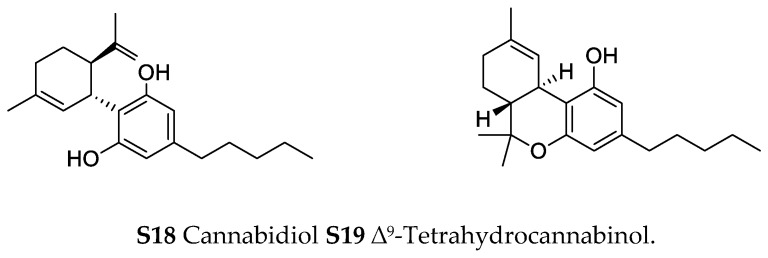
Cannabinoids **S18** and **S19**.

In the genus *Orobanche* s.l. the following classes of secondary metabolites have been found: aromatic aldehydes, ketones, and phenylmethanoids (Figure 1), phenylethanoids (Figure 2), phenylethanoid glycosides (Figure 3), phenylpropanoid glycosides (Figure 4, Figure 5, Figure 6, Figure 7 and Figure 8), phenolic acids (Figure 9 and Figure 10), lignans (Figure 11 and Figure 12), flavonoids (Figure 13), a tropone derivative (Figure 14), and sterols (Figure 15, Figure 16, Figure 17, Figure 18, Figure 19 and Figure 20). Phenylpropanoid glycosides represent the largest group of secondary metabolites isolated from *Orobanche* species. Moreover, bi- and tricyclic sesquiterpenes, iridoid glycosides, acyclic, monocyclic, and bicyclic monoterpenes, and carotenoids have been detected in *Orobanche*. Along with secondary metabolites the isolation of some primary metabolites such as fatty acids, alkanes, alkenes, ketones, fatty alcohols, and sugar alcohols has been described. Species that have been investigated for their phytochemical compounds are subsequently listed in alphabetical order within their corresponding genus. The secondary metabolites synthesized by *Orobanche* species are shown in Figures 1–20 and compounds are numbered consecutively from **1** to **70**. Components of essential oils are listed in the corresponding paragraphs but individual chemical structures are neither displayed nor numbered. Primary metabolites that have been isolated along with secondary metabolites and that are mentioned in the corresponding papers are only mentioned in the text (no figure, no numbers). Secondary metabolites sequestered from host species by *Orobanche* species, comprising alkaloids (Figure 21, Figure 22, Figure 23 and Figure 24), polyacetylenes (Figure 25), and cannabinoids (Figure 26), are shown in Figure 21, Figure 22, Figure 23, Figure 24, Figure 25 and Figure 26, and these sequestered compounds are numbered consecutively from **S1** to **S19**. Information about collection site, country of origin, analyzed plant parts, and extraction solvents as well as analytical methods used for compound identification and structure elucidation are listed in this order in brackets after the corresponding taxon. Analytical methods include infrared spectroscopy (IR), gas chromatography (GC), gas liquid chromatography (GLC), mass spectrometry (MS), nuclear magnetic resonance spectroscopy (NMR), thin layer chromatography (TLC), high performance thin layer chromatography (HPTLC), and high performance liquid chromatography (HPLC) with ultraviolet (UV) and diode array detectors (DAD). Most authors do not specify the host species of the investigated *Orobanche* samples. Whenever available, information about the host species is mentioned. An additional section is dedicated to reports dealing with more than one *Orobanche* species. Natural products described in these reports are also briefly mentioned in the respective paragraphs for each individual species. An additional section is dedicated to the sequestration of secondary metabolites by *Orobanche* species from their host plants. Moreover, one section deals with bioactivities reported for *Orobanche* extracts and natural products isolated from such extracts. Synonyms of taxon and compound names are only indicated when being mentioned for the first time. Tables giving an overview of all phytochemically investigated species, natural products detected within these species and sequestered by these species, respectively, are available as Supplementary Material (Tables S1 and S2).

### 2.2. Secondary Metabolites Synthesized by Orobanche s.l.

#### 2.2.1. *Orobanche* Sectio *Orobanche* (=sect. *Osproleon* Wallr. = Genus *Orobanche* L. s.str.)

***Orobanche alba* Stephan ex Willd.**—Roudbaraki and Nori-Shargh identified forty different compounds in the essential oil from *O. alba* [province of Guilan, Iran; aerial parts; hydrodistillation; GC, GC-MS, comparison with authentic samples] [host species not mentioned] [11]. Detected compounds included three monoterpene hydrocarbons, twelve oxygenated monoterpenes, five sesquiterpene hydrocarbons, three oxygenated sesquiterpenes, one oxygenated diterpene, and sixteen non-terpenic compounds (aliphatic hydrocarbons, alcohols, ethers, aldehydes, ketones, carboxylic acids, and esters). The monoterpene fraction encompassed bornylangelate, *p*-cymene, limonene, γ-terpinene, *p*-menthone, 1,8-cineol, pinocamphone, linalool, (*Z*)-iso-citral, nerol, neral (syn. citral B, *cis*-isomer), geraniol, geranial (syn. citral B, *trans*-isomer), and geranylacetate; the sesquiterpene fraction included *trans*-caryophyllene (syn. β-caryophyllene), 6,9-guaiadiene, isobornyl-2-methyl-butyrate, δ-cadinene α-copaene, β-bourbonene, and caryophyllene oxide. The only diterpene detected was manool and the only phenylpropanoid methyl chavicol (syn. estragol). Detected non-terpene compounds comprised *n*-nonanal, neryl acetate, myristic acid, palmitic acid, linoleic acid, linolenic acid, 6-methyl-5-hepten-2-one, 6,10,14-trimethyl-2-pentadecanone, octadecane, heneicosane, docosane, tricosane, tetracosane, pentacosane, nonadecene, isobutyl phthalate, and bis(2-ethylhexyl)phthalate. Fruchier et al. reported the isolation of the tropone derivative orobanone (**45**) from *O. alba* (as *O. epithymum* DC.) [whole plants; water, chloroform; IR, UV, MS, CI-MS, ^1^H and ^13^C NMR] [host species not mentioned] [12].

***Orobanche amethystea* Thuill.**—In *O. amethystea* Serafini et al. [Sardinia, Italy; flowering plants; alcoholic extract; ^1^H and ^13^C NMR, HPLC, co-elution of extracts with isolated and identified phenylpropanoid glycosides] [host species not mentioned] detected the phenylpropanoid glycosides verbascoside (syn. acteoside, orobanchin, [13]) (**10**) and oraposide (syn. crenatoside and orobanchoside) (**29**) [14].

***Orobanche anatolica* Boiss. & Reut.**—The occurrence of saponins in *O. anatolica* was mentioned by Aynehchi et al. [Iran; whole plant] [host species not mentioned] but no further specification of structures nor of any analytical methods were indicated in the report [15].

***Orobanche artemisiae-campestris* Gaudin subsp. *picridis* (F.W.Schultz) O. Bolòs, Vigo, Masalles & Ninot**—Fruchier et al. isolated the tropone derivative orobanone (**45**) [whole plant; water, chloroform; IR, UV, MS, CI-MS, ^1^H and ^13^C NMR] [no information about the host] from *O. artemisiae-campestris* subsp. *picridis* (using the synonym *O. picridis* F.W.Schultz) [12].

***Orobanche caryophyllacea* Sm.**—The tropone derivative orobanone (**45**) was isolated from *O. caryophyllacea* (using the synonym *O. major* L.) by Fruchier et al. [whole plant; water, chloroform; IR, UV, MS, CI-MS, ^1^H and ^13^C NMR] [no information about the host] [12].

***Orobanche cernua* Loefl.**—Qu et al. characterized 17 compounds from *O. cernua* (syn. *O. cumana* Wallr., *O. cernua var. cumana* Wallr.) [Jilin province, China; fresh whole plants; methanol; MS, 1D and 2D NMR, comparison with literature data] [host species not mentioned] [17]. Eleven compounds were identified as phenylpropanoid glycosides [salidroside (**8**), acteoside (**10**), 2′-*O*-acetylacteoside (**14**), campneoside (**18**), leucosceptoside A (**19**), isoacteoside (**23**), isocampneoside (**24**), oraposide (**29**), 3‴-*O*-methylcrenatoside (**30**), descaffeoyl crenatoside (**31**), and isocrenatoside (**32**)]. Furthermore, three phenolic acids [caffeic acid (**34**), *trans*-ferulic acid (**36**), and chlorogenic acid (**37**)], one lignan [dimethyl-6,9,10-trihydroxybenzol[*kl*]xanthene-1,2-dicarboxylate (**38**)], and two flavonoids [apigenin (**42**) and luteolin (**43**)], were found. In a second report Qu et al. described the isolation of a novel phenylethanoid glycoside, 3′-*O*-methylisocrenatoside (**33**) as well as of the known compounds protocatechuic aldehyde (**2**) and methyl caffeate (**35**) from *O. cernua* [Jilin Province, China; fresh whole plant; methanol; IR, MS, NMR] [host species not mentioned] [18]. Yang et al. examined *O. cernua* [Neimenggu province, China; whole plant; ethanol 70%, reflux; HPLC-MS, NMR] [host species not mentioned] isolating twelve compounds: eight phenylpropanoid glycosides [acteoside (**10**), campneoside II (**17**), campneoside I (**18**), leucosceptoside A (**19**), isoacteoside (**23**), cistanoside F (**27**), oraposide (**29**), and isocrenatoside (**32**)]; three lignans [(+)-pinoresinol 4′-*O*-β-D-glucopyranoside (**39**), isoeucommin A (**40**), and (+)-syringaresinol 4′-*O*-β-D-glucopyranoside (**41**)], and one steroid [stigmasterol 3-*O*-β-D-glucoside (**54**)] [19].

***Orobanche coerulescens* Stephan ex Willd.**—Zhao et al. isolated the phenylpropanoid glycoside acteoside (**10**) from *O. coerulescens* [Xinjiang province, China; ethanol] [host species not mentioned] [20]. The isolation of isoacteoside (**23**), sinapoyl 4-*O-*β-D-glucoside (**26**), cistanoside F (**27**), oraposide (**29**), and adenosine was described in a second report [21] [Xinjiang province, China; rhizome; ethanol 95%, reflux; TLC, NMR] [host species not mentioned]. Acteoside (**10**), cistanoside F (**27**), and oraposide (**29**) were also isolated by Murayama et al. along with 2-phenylethyl β-primeveroside (**7**), rhodioloside (syn. salidroside) (**8**), descaffeoyl crenatoside (**31**), and isocrenatoside (**32**) from *O. coerulescens* [Niigata prefecture, Japan; whole plant; methanol; IR, UV, HR-FAB-MS, ^1^H,^1^H-DQF, COSY, ^1^H,^1^H-relayed COSY, HMQC, and HMBC NMR] [host species not mentioned] [22]. Lin et al. isolated two new phenylpropanoid glycosides, caerulescenoside (**13**) and 3′-methyl crenatoside (**30**), along with five known phenylpropanoid glycosides, desrhamnosyl acteoside (**9**), acteoside (**10**), campneoside (**18**), isoacteoside (**23**), and oraposide (**29**) from *O. coerulescens* [Taipei, Taiwan; whole plant; ethanol 95%; IR, UV, ^1^H NMR, ^13^C NMR, ESI-MS, FAB-MS, HR-FAB-MS] [host species not mentioned] [23]. In a second report the authors mentioned the occurrence of another phenylpropanoid glycoside, rossicaside B (**28**), in *O. coerulescens*; ethanol 95%, preparation in accordance with former report, no information about analytical methods) [24]. Wang et al. also isolated a new phenylpropanoid glycoside from the whole plant of *O. coerulescens* [Neimenggu province, China; whole plant; ethanol 50%; TLC, HPLC, MS, NMR] [host species not mentioned] [25]. The structure was identified as 2-(3-methoxy-4-hydroxy)-phenyl-ethanol-1-*O*-α-L-[(1→3)-4-*O*-acetyl-rhamnopyranosyl-4-*O*-feruloyl]-*O*-β-D-glucopyranoside and named orobancheoside A (**22**). Additionally, Zhao et al. described the isolation protocatechuic aldehyde (**2**) and caffeic acid (**34**) as well as of β-sitosterol (**50**) and daucosterol (**53**) from *O. coerulescens* [Xinjiang province, China; rhizome; ethanol 95%, reflux; NMR] [host species not mentioned] [26]. Shao et al. identified the phenylpropanoid glycosides acteoside (**10**) and oraposide (**29**), as well as sterols β-sitosterol (**50**), stigmasterol (**51**), and β-daucosterol (**53**) [Neimenggu province, China; ethanol 95%, reflux; TLC, MS, NMR] [host species not mentioned] [27]. Moreover, primary metabolites D-mannitol, glyceryl arachidate, succinic acid, and D-pinitol were reported. Recently, Zhang characterized a new phenethyl alcohol glycoside named orobancheoside B (**21**) from *O. coerulescens* [Neimenggu province, China; whole plant; ethanol 50%; IR, UV, MS, NMR] [host species not mentioned] [28].

***Orobanche crenata* Forssk.**—El-Shabrawy et al. found two phenylpropanoid glycosides in *O. crenata* growing on *Vicia faba* L. [Egypt; chloroform for removal of non-polar compounds, aqueous ethanol 70%; TLC, NMR] [29]. However, their structures were not fully characterized. Afifi et al. extracted secondary metabolites from *O. crenata* parasitizing on *V. faba* [Mansoura, Egypt; aerial parts; ethanol 90%; melting point, TLC, IR, UV, ^1^H NMR, ^13^C NMR, ^13^C NMR-DEPT, FAB-MS] and isolated the known phenylpropanoid glycoside acteoside (**10**) as well as the new phenylpropanoid glycoside oraposide (**29**) [30]. Acteoside (**10**) was also extracted by Gatto et al. [parasitizing on *V. faba*; Apulia, Italy; stems; methanol 80% under reflux; comparison of UV spectra and retention times with standard substances, HPLC-DAD] who furthermore found isoverbascoside (syn. isoacteoside; an isomer of verbascoside) (**23**) and an unidentified caffeic acid derivative [31,32,33]. Serafini et al. isolated verbascoside (**10**), poliumoside (**11**), and orobanchoside (**29**) from *O. crenata* [Sardinia, Italy; flowering plants; alcoholic extract; ^1^H and ^13^C NMR, HPLC, co-elution of extracts with isolated and identified phenylpropanoid glycosides] [host species not mentioned] [14]. Orobanchoside (**29**) was proven to be structurally identical with oraposide and oraposide by Nishibe et al. in the same year [34]. Nada and El-Chaghaby analyzed ethanolic (80%) extracts of *O. crenata* grown on *V. faba* by GC-MS and postulated the occurrence of 6-monohydroxyflavone, glycitein, actinobolin, hexestrol, and 2,4-di-tert-butylphenyl benzoate [Egypt; ethanol 80%; GC-MS] [35]. However, none of these compounds are considered any further here, because the data on the used GC system are incomplete and data on peak identification procedures are completely missing. Until contrary evidence will have been procured, we do not consider 6-monohydroxyflavone, glycitein, actinobolin, hexestrol, and 2,4-di-tert-butylphenyl benzoate natural products which have been detected in the genus *Orobanche*. Dini et al. investigated the phytochemistry of *O. crenata* using the synonym *O. speciosa* DC. [Molise, Italy; aerial parts; petroleum ether, chloroform, methanol; comparison of UV, IR, ^1^H and ^13^C NMR spectral data with literature data] [36]. The three phenylpropanoid glycosides verbascoside (**10**), poliumoside (**11**), and oraposide (**29**) were detected. Along with these compounds the authors also isolated *p*-hydroxy benzaldehyde (**1**), isovanillin (**3**), vanillin (**4**), syringaldehyde (**5**), and *p*-hydroxy acetophenone (**6**). Fruchier et al. reported the isolation of the tropone derivative orobanone (**45**) from *O. crenata* in their investigation of the occurrence of this secondary metabolite in various *Orobanche* species [whole plant; water, chloroform; IR, UV, MS, CI-MS, ^1^H and ^13^C NMR] [host species not mentioned] (see extra paragraph and supplementary material) [12]. Abbes et al. described the extraction of polyphenols and tannins from *O. crenata* growing on *V. faba* without any further specification of the substances [Beja and Ariana Governorates, Tunisia; aerial parts; methanol; water] [37].

***Orobanche denudata* Moris**—Serafini et al. isolated the phenylpropanoid glycosides verbascoside (**10**) and orobanchoside (**29**) from *O. denudata* [Sardinia, Italy; flowering samples; alcoholic extract; ^1^H and ^13^C NMR, HPLC, co-elution of extracts with isolated and identified phenylpropanoid glycosides] [host species not mentioned] [14].

***Orobanche foetida* Poir.**—Abbes et al. described the extraction of polyphenols and tannins from *O. foetida* growing on *V. faba* without any further specification of the substances [Beja and Ariana Governorates, Tunisia; aerial parts; methanol; water] [37].

***Orobanche gracilis* Sm.**—Fruchier et al. isolated the tropone derivative orobanone (**45**) from *O. gracilis* (using the synonym *O. cruenta* Bertol.) [whole plants; water, chloroform; IR, UV, MS, CI-MS, and ^1^H and ^13^C NMR] [no information about the host] [12].

***Orobanche grisebachii* Reut.**—Aynilian et al. screened several *Orobanche* species for their contents of alkaloids, tannins, and saponins, without reporting any particular structures of the metabolites. *O. grisebachii* [plant material obtained from The Post Herbarium of the American University of Beirut, Lebanon; ethanol 95%, ethanol 80%] contained alkaloids and tannins [40].

***Orobanche hederae* Duby**—Pieretti et al. isolated the phenylpropanoid verbascoside (**10**) and orobanchoside (**29**) from *O. hederae* [Lazio, Italy; whole plants; ethanol; ^1^H NMR, HPLC-UV] [host species not mentioned] [41]. Capasso et al. also found these two compounds [whole plants; ethanol] [host species not mentioned] [42]. As well as in eleven other species of the genus *Orobanche* (see extra paragraph) Fruchier et al. found the tropone derivative orobanone (**45**) in *O. hederae* [whole plant; water, chloroform; IR, UV, MS, CI-MS, ^1^H and ^13^C NMR] [host species not mentioned] [12]. Baccarini and Melandri isolated and analyzed seven pigments from *O. hederae* growing on *Hedera helix* L. (Araliaceae) [whole plant; acetone 80%; TLC, comparison with standard substances]. These pigments were β-carotene, α-carotene-5,6-epoxide, flavochrome, lutein-5,6-epoxide, flavoxanthin, and taraxanthin [43]. The seventh compound was tentatively identified as neoxanthin. There is also a report of the sequestration of substances from its host species by *O. hederae*. This is described in a separate paragraph below.

***Orobanche loricata* Rchb.**—The isolation of the phenylpropanoid glycosides verbascoside (**10**) and orobanchoside (**29**) from *O. loricata* was described by Serafini et al. [Sardinia, Italy; flowering samples; alcoholic extract; ^1^H and ^13^C NMR, HPLC, co-elution of extracts with isolated and identified phenylpropanoid glycosides] [host species not mentioned] [14]. Fruchier et al. isolated the tropone derivative orobanone (**45**) [whole plants; water, chloroform; IR, UV, MS, CI-MS, ^1^H and ^13^C NMR] [no information about the host] [12].

***Orobanche lutea* Baumg.**—Rohmer et al. analyzed several parasitic species for their sterol contents and sterol biosynthesis [44]. The following compounds were isolated and identified from *O. lutea* [Alsace, France; stems; acetone, chloroform:methanol (2:1); GLC, GLC-MS] [host species not mentioned]: sterols [stigmastanol (**46**), cholesterol (**47**), campesterol (**48**), 24-methylene cholesterol (**49**), sitosterol (**50**), stigmasterol (**51**), *iso*fucosterol (**52**), ∆^7^-campestenol (**55**), episterol (**56**), ∆^7^-stigmastenol (**57**), and ∆^7^-avenasterol (**58**)], 4α-methylsterols [24- 24-methyl lophenol (**59**), methylene lophenol (**60**), 24-ethyl lophenol (**61**), 24-ethylidene lophenol (**62**), 4α-methyl ∆^8^-campestenol (**63**), 4α-methyl ∆^8^-stigmastenol (**64**), 24,28-dihydro obtusifoliol (**65**), obtusifoliol (**66**), 24,28-dihydrocycloeucalenol (**67**), cycloeucalenol (**68**), and 4,4-dimethylsterols cycloartenol (**69**)], and 24-methylene cycloartenol (**70**).

***Orobanche minor* Sm.**—Kurisu et al. isolated the phenylpropanoid glycosides acteoside (**10**) and oraposide (**29**) from *O. minor* [whole plant; methanol; NMR (^1^H, ^13^C, COSY, HMQC, HMBC, NOESY), HR-ESI-MS] [host species not mentioned] [45]. Serafini et al. also extracted verbascoside (**10**) and orobanchoside (**29**) [Sardinia, Italy; flowering samples; alcoholic extract; ^1^H and ^13^C NMR, HPLC, co-elution of extracts with isolated and identified phenylpropanoid glycosides] [host species not mentioned] [14]. Kidachi et al. isolated acteoside (**10**), cistanoside D (**20**), isoacteoside (**23**), oraposide (**29**), 3‴-*O*-methyl crenatoside (**30**), and isocrenatoside (**32**) [whole plant; methanol] [host species not mentioned] [46]. The tropone derivative orobanone (**45**) was found in *O. minor* by Fruchier et al. [whole plant; water, chloroform; IR, UV, MS, CI-MS, ^1^H and ^13^C NMR] [host species not mentioned] [12].

***Orobanche owerinii* Beck**—Dzhumyrko and Sergeeva detected several carotenoids in *O. owerinii* [epigeal parts; *n*-hexane, petrol ether; co-chromatography with reference compounds and UV spectroscopy] growing on the hypogeal organs of *Fraxinus* [47]. Violaxanthin, auroxanthin, the ester of violaxanthin and palmitic acid, as well as α- and β- carotenes were detected.

***Orobanche pubescens* d´Urv.**—Aynilian et al. screened several *Orobanche* species for their contents of alkaloids, tannins, and saponins, without investigating any particular structures of the metabolites. Tannins were found in *O. pubescens* (using the synonym *O. versicolor* F.W.Schultz) [plant material obtained from The Post Herbarium of the American University of Beirut, Lebanon; petroleum benzine (defatting), ethanol 95%, ethanol 80%] [40].

***Orobanche pycnostachya* Hance**—Han et al. isolated eight compounds from *O. pycnostachya* [Neimenggu province, China; ethanol 95%; MS, NMR] [host species not mentioned] [48]. The primary metabolites *n*-nonacosane acid, *n*-hexacosyl alcohol, *D*-allitol, 2,3,4,6-α-D-galactopyranose tetramethyl ether, and secondary metabolites acteoside (**10**), fissistigmoside (**25**), β-sitosterol (**50**), and daucosterol (**53**) were identified. Li et al. also isolated the phenylpropanoid glycosides acteoside (**10**) as well as 2′-*O*-acetylacteoside (**14**), and oraposide (**29**) [Mengu, Anhui and Hebei, China; methanol 70%; HPLC] [host species not mentioned] [49].

***Orobanche rapum-genistae* Thuill.**—Several secondary metabolites such as phenylproanoid glycosides, alkaloids, and a tropone derivative have been isolated from *O. rapum-genistae*. The isolated alkaloids are presumably not synthesized by the parasite itself but sequestered from the host species. Sequestration of secondary metabolites by *O. rapum-genistae* from its host is also reported by other authors and described in a separate paragraph below. Fruchier et al. isolated the tropone derivative orobanone (**45**) from *O. rapum-genistae* parasitizing on *Cytisus scoparius* (L.) Link and *Cytisus purgans* (L.) Spach (both Fabaceae) [whole plant; water, chloroform; IR, UV, MS, CI-MS, ^1^H and ^13^C NMR] [12]. Eleven other *Orobanche* species were also screened for orobanone (**45**) (see separate paragraph and supplementary material). Several sources report the isolation of the two phenylpropanoid glycosides verbascoside (**10**) and oraposide (**29**) from *O. rapum-genistae* (Andary et al., [50,51] (host: *C. scoparius*, *C. purgans*), [39] [phenolic extract; hydrolysis, high resolution ^1^H and ^13^C NMR], [38] [^1^H and ^13^C NMR, X-ray crystal analysis]; Bridel and Charaux, [52,53] [tubers/bulbs; alcohol]; Serafini et al., [14] [Sardinia, Italy; flowering plants; alcoholic extract; ^1^H and ^13^C NMR, HPLC, co-elution of extracts with isolated and identified phenylpropanoid glycosides] [host species not mentioned]; Viron et al., [54] [whole plant; aqueous ethanol 70%; HPTLC, HPLC] [host species not mentioned], [55]). Andary et al. described the differentiation of two ecotypes of *O. rapum-genistae*: *O. rapum-cytisi scoparii* (parasitizing *C. scoparius*) and *O. rapum-cytisi purgantis* (parasitizing *C. purgans*) based on morphological and phytochemical characteristics [51]. Phenylpropanoid glycosides verbascoside (**10**) and orobanchoside (**29**) as well as quinolizidine alkaloids sparteine (**S2**) and lupanine (**S4**) were found in both ecotypes in different concentrations.

***Orobanche sanguinea* C.Presl.**—Serafini et al. isolated the phenylpropanoid glycosides verbascoside (**10**) and orobanchoside (**29**) from *O. sanguinea* [Sardinia, Italy; flowering samples; alcoholic extract; ^1^H and ^13^C NMR, HPLC, co-elution of extracts with isolated and identified phenylpropanoid glycosides] [host species not mentioned] [14].

***Orobanche variegata* Wallr.**—Fruchier et al. isolated the tropone derivative orobanone (**45**) from *O. variegata* [whole plant; water, chloroform; IR, UV, MS, CI-MS, ^1^H and ^13^C NMR] [no information about the host] [12].

#### 2.2.2. Phelipanche (=Orobanche Sectio Trionychon)

***Phelipanche aegyptiaca* (Pers.) Pomel** (formerly ***O. aegyptiaca* Pers.**)—Afifi et al. isolated acteoside (**10**), poliumoside (**11**), 2′-*O*-acetylacteoside (**14**), and 2′-*O*-acetylpoliumoside (**15**) from *P. aegyptiaca* (as *O. aegyptiaca*) [butanol extraction] [host species not mentioned [56]. Sharaf and Youssef described the extraction of alkaloids and tannins (organic acids, reducing sugars, glucosides resins, and unsaturated substances) from *P. aegyptiaca* (as *O. aegyptiaca*) [whole plant; 30% aqueous extract, hexane, chloroform, ethyl alcohol] [host species not mentioned] [57]. The extracted substances were not characterized any further.

***Phelipanche arenaria* Pomel (Syn.: *O. arenaria* Borkh.)**—Andary et al. isolated the caffeic acid glycosides arenarioside (**12**) and pheliposide (**16**) from *P. arenaria* (as *O. arenaria*) growing on *Artemisia campestris* Ledeb. var. *glutinosa* (J.Gay ex Besser) Y.R.Ling [syn. of *A. campestris* subsp. *glutinosa* (Besser) Batt.] [Hérault, France; ethanol 80%; high resolution ^1^H and ^13^C NMR]. Orobanone (**45**) was found in *P. arenaria* (as *O. arenaria*) by Fruchier et al., (1981) [whole plant; water, chloroform; IR, UV, MS, CI-MS, ^1^H and ^13^C NMR] [host species not mentioned] [12].

***Phelipanche oxyloba* (Reut.) Soják** (Syn.: ***O. oxyloba* (Reut.) Beck**)—Aynilian et al. screened several *Orobanche* species for their contents of alkaloids, tannins, and saponins, without reporting fully characterized metabolites. Tannins were found in *P. oxyloba* (as *O. nana* Noe ex G.Beck) [plant material obtained from The Post Herbarium of the American University of Beirut, Lebanon; petroleum benzine (defatting), ethanol 95%, ethanol 80%] [40].

***Phelipanche ramosa* (L.) Pomel** (Syn.: ***O. ramosa* L.)**—Lahloub et al. extracted known compounds acteoside (**10**) and 2′-*O*-acetylacteoside (**14**) as well as the formerly undescribed phenylpropanoid glycoside 2′-*O*-acetylpoliumoside (syn. brandioside, [14]) (**15**) from *Phelipanche ramosa* (using the synonym *Orobanche ramosa*) parasitizing on *Lycopersicon esculentum* Mill. [El-Behera Governorate, Egypt; whole plant; percolation in ethanol; comparison of melting point, IR, UV, ^1^H and ^13^C NMR with literature data] [58]. Serafini et al. isolated the phenylpropanoid glycosides verbascoside (**10**) and orobanchoside (**29**) from *P. ramosa* subsp. *ramosa* (as *O. ramosa* subsp. *ramosa*) and *P. nana* (Reut.) Soják (as *O.ramosa* subsp. *nana* (Reut.) Cout., syn. *O. nana* (Reut.) Beck, according to [10]) as well as poliumoside (**11**) from *P*. *nana* [Sardinia, Italy; flowering samples; alcoholic extract; ^1^H and ^13^C NMR, HPLC, co-elution of extracts with isolated and identified phenylpropanoid glycosides] [host species not mentioned] [14]. The tropone derivative orobanone (**45**) was isolated from *P. ramosa* by Fruchier et al. (as *O. ramosa*) [whole plant; water, chloroform; IR, UV, MS, CI-MS, ^1^H and ^13^C NMR] [host species not mentioned] [12]. Afifi et al. also found poliumoside (**11**) in *P. ramosa* (as *O. ramosa*) [butanol extraction] [host species not mentioned] [56]. Two papers report tricin (**44**) (5,7,4′-trihydroxy-3,5′-dimethoxyflavone) from the seeds and aerial parts of *P. ramosa* (as *Orobanche ramosa*), respectively [59,60]. Compound identification [59]: spectra of the pigments and their acetates, R_f_ values, color properties on paper chromatograms and mixed melting points; [60]: UV and MS analyses, demethylation to tricetin and column chromatography]; host species not mentioned. Sequestration of secondary metabolites from its host by *P. ramosa* is described in a separate section below.

#### 2.2.3. Reports Describing the Investigation of more than One Species

Besides investigating the phytochemical composition of one species there are also several reports dealing with more than one species. Serafini et al. investigated the secondary metabolite contents of several *Orobanche* species [Sardinia, Italy; flowering samples; alcoholic extract; ^1^H and ^13^C NMR, HPLC, co-elution of extracts with isolated and identified phenylpropanoid glycosides] [host species not mentioned] [14].

The authors reported the occurrence of the phenylpropanoid glycosides verbascoside (**10**) and orobanchoside (**29**) in *O. amethystea*, *O. crenata*, *O. denudata*, *O. hederae*, *O. loricata*, *O. minor*, *O. rapum-genistae* subsp. *rigens* (Loisel.) Arcang. (as *O. rigens* Loisel. [10]), *O. sanguinea*, *P. nana* (as *O. ramosa* subsp. *nana*), and *P. ramosa* subsp. *ramosa* (as *O. ramosa* subsp. *ramosa*). In *O. crenata* and *P*. *nana* poliumoside (**11**) was additionally reported. Moreover, the isolation of verbascoside (**10**), 2′-*O*-acetylacteoside (**14**), and 2′-*O*-acetylpoliumoside (**15**) from *P. ramosa* (as *O. ramosa*) [58], and pheliposide (**16**) and orobanchoside (**29**) from *O. arenaria* [61] were mentioned in the report from Serafini et al. [14]. The reference about orobanchoside (**29**) occurring in *O. arenaria* by Andary et al. [61] is questionable because in the named report arenarioside (**12**) is described instead of orobanchoside (**29**). Fruchier et al. describe the isolation of the guaian type sesquiterpene tropone derivative orobanone (3,8-dimethyl-*S*-isopropyl-2,3-dihydro(lH)azulen-6-one) from *O. rapum-genistae* parasitizing on *C. scoparius* and *C. purgans* [whole plant; water, chloroform; IR, UV, MS, CI-MS, ^1^H and ^13^C NMR] [12]. No traces of orobanone were found in the hosts. This compound was also found in eleven other *Orobanche* species: *O. alba* (as *O. epithymum*), *O. arenaria*, *O. artemisiae-campestris* subsp. *picridis* (as *O. picridis*), *O. caryophyllacea* (as *O. major*), *O. crenata*, *O. gracilis* (as *O. cruenta*), *O. hederae*, *O. loricata*, *O. minor*, *O. ramosa*, and *O. variegata*. Except for *O. rapum-genistae*, no information about the host species were indicated.

Aynilian et al. [plant material obtained from The Post Herbarium of the American University of Beirut, Lebanon; petroleum benzine (defatting), ethanol 95%, ethanol 80%] screened several *Orobanche* species for their contents of alkaloids, tannins, and saponins without reporting any defined chemical compounds [40]. Tannins were found in all six analyzed species, *O. aegyptiaca*, *O. crenata*, *O. grisebachii*, *O. oxyloba* (as *O. nana*), *O. pubescens* (as *O. versicolor*), and *P. ramosa* (as *O. ramosa*). Saponins were absent in all investigated taxa, and alkaloids were detected in *O. crenata*, *O. grisebachii*, *O. pubescens*, and *O. ramosa*.

### 2.3. Sequestration of Secondary Metabolites by Orobanche s.l. from Their Host Species

As obligate holoparasites *Orobanche* species drain water and essential nutrients from their host plants. Also the sequestration bioactive natural products from the hosts seems likely [62], but up to now little is known about the uptake of other substances by holoparasitic *Orobanche* species from the plants they parasitize on and only very few reports deal with the sequestration of secondary metabolites from hosts.

#### 2.3.1. *Orobanche* Section *Orobanche* (=sect. *Osproleon* Wallr. = Genus *Orobanche* L. s.str.)

***Orobanche hederae* Duby**—Sequestration of minerals and fatty acids by *Orobanche hederae* from its host *Hedera helix* was described by Lotti and Paradossi [Tuscany, Italy; whole plants; petrol ether, soxhlet; GC [63,64]. Sareedenchai and Zidorn reported the uptake of polyacetylenes falcarinol (**S15**), 11,12-dehydrofalcarinol (**S16**) and 11,12,16,17-didehydrofalcarinol (**S17**) from the roots of *Hedera helix* by *Orobanche hederae* [Trentino-Alto Adige, Italy; whole plants; dichloromethane; HPLC-DAD, HPLC-MS, comparison with authentic reference compounds and literature data] [65].

***Orobanche rapum-genistae* Thuill.**—Wink et al. reported *O. rapum-genistae* sequestering quinolizidin alkaloids N-methylangustifoline (**S1**), sparteine (**S2**), 17-oxosparteine (**S3**), lupanine (**S4**), 17-oxolupanine (**S5**), hydroxylupanine (**S6**), 13-tigloyloxylupanine (**S7**), 13-angeloyloxylupanine (**S8**), 13-benzoyloxylupanine (**S9**), 4-hydroxylupanine (**S10**), anagyrine (**S11**), 13-5,6-dehydrolupanine (**S12**), and dehydrosparteine (**S13**) as well the piperidine alkaloid ammodendrine (**S14**) from *Cytisus scoparius* [as *Sarothamnus scoparius* (L.) W.D.J. Koch] [Rhineland-Palatinate, Germany; stems, leaves, pods, seeds, bulbs, shoots; HCl, alkalization, Extrelut-column, dichloromethane; GLC/GLC-MS, comparison with retention indices of known reference alkaloids] [66]. From the report it is not clearly visible whether 13-tigloyloxylupanine (**S7**), 13-angeloyloxylupanine (**S8**), 13-benzoyloxylupanine (**S9**), and 4-hydroxylupanine (**S10**) are present in the α- or the β-form, respectively. Rascol et al. also described the isolation of quinolizidine alkaloids (−)-sparteine (**S2**), (+)-lupanine (**S4**), and (+)-13-hydroxylupanine (**S6**) from *O. rapum-genistae* growing on *Cytisus* [67]. In a second report the authors investigated the effects of alkaloid contents of the host (*Cytisus scoparius* and *Cytisus purgans*) on the alkaloid contents of the parasite and vice versa [68].

#### 2.3.2. Phelipanche (=Orobanche Section Trionychon)

***Phelipanche ramosa* (L.) Pomel** (syn.: ***Orobanche ramosa* L.**)—Cannabinoids cannabidiol (**S18**) and ∆^9^-tetrahydrocannabinol (**S19**) were found by Fournier and Paris in traces in *Orobanche ramosa* growing on *Cannabis sativa* L. (Cannabaceae) [petrolether; TLC, GC-MS] [69].

### 2.4. Sequestration of Secondary Metabolites from Host Plant Species by Other Hemiparasitic and Holoparasitic Plant Taxa

Sequestration is defined as the uptake, accumulation, and eventual use of substances, especially toxins, by animals or plant species from other organisms (microbes, plants or other animals) [70]. Most reports deal with interactions between plants and herbivorous insects, but sequestration is also known from some gastropods (feeding on e.g., algae, sponges, and bacteria) as well as from hemiparasitic and holoparasitic plants [71]. In most reported cases toxic secondary plant metabolites such as cardenolides, iridoid glycosides, and pyrrolizidine alkaloids or their precursors are sequestered by herbivorous insects and utilized as a defense against predators. Insects actively sequestering toxic compounds have, during their evolution, developed mechanisms to maintain the chemical stability of the sequestered toxins, while tolerating these toxins, without being negatively affected by their toxicity [72,73]. Sequestered metabolites can play a role in reproduction when utilized as sex pheromones [74]. There are also reports of sequestration of non-toxic substances like carotenoids or flavonoids by herbivorous insects [75]. Besides plant-animal interactions, some authors describe plant-plant interactions. Mainly alkaloids, but also iridoid glycosides, cardenolides, cardiac glycosides, and glucosinolates have been described to be sequestered. Most of the parasitic taxa analyzed so far are hemiparasites. Sequestration of iridoid glycosides by the hemiparasite *Euphrasia stricta* J.F.Lehm (Orobanchaceae, formerly Scrophulariaceae) from its host *Galium verum* L. (Rubiaceae) [76], and by the hemiparasite *Castilleja integra* A.Gray (Orobanchaceae) from *Penstemon teucrioides* Greene (Plantaginaceae) were described [77]. Pyrrolizidine and quinolizidine alkaloids are sequestered by hemiparasitic *Castilleja* Mutis ex L.f. species (Orobanchaceae, formerly Scrophulariaceae) from their hosts *Senecio atratus* Greene and *Senecio triangularis* Hook. (both Asteraceae), *Lupinus* L. species (Fabaceae), and *Thermopsis montana* Torr. & A.Gray (Fabaceae) [78,79,80]. Quinolizidine alkaloids are sequestered by *Orthocarpus* Nutt. species (Orobanchaceae) from *Lupinus* species, and by holoparasitic *Cuscuta* L. species (Convolvulaceae) from *Genista acanthoclada* DC. (Fabaceae), *Lupinus*, *Cytisus* L., and *Spartium* L. species (all Fabaceae) as well as from several other Fabaceae species [81,82,83]. Hemiparasitic *Pedicularis* L. species (Orobanchaceae, formerly Scrophulariaceae) were reported to sequester alkaloids from *Senecio* L. and *Thermopsis* R.Br. species as well as from *Picea engelmannii* Parry ex Engelm. (Pinaceae) [84]. The uptake of norditerpenoid alkaloids by *Castilleja sulphurea* Rydb. from *Delphinium* L. species (Ranunculaceae) was described. Hemiparasitic *Tristerix verticillatus* (Ruiz & Pav.) Barlow & Wiens (Loranthaceae) was found to take up isoquinoline alkaloids from *Berberis montana* Gay (Berberidaceae), and bipiperidyl and quinolizidine alkaloids synthesized by *Retama sphaerocarpa* (L.) Boiss. and *Lygos sphaerocarpa* (L.) Heyw. (both Fabaceae) were found to be sequestered by hemiparasitic *Viscum cruciatum* Sieber ex Boiss. (Santalaceae) [85,86,87,88,89]. An interesting study of community-level interactions described the transfer of alkaloids produced by an endophytic fungus [*Neotyphodium uncinatum* (W. Gams, Petrini & D. Schmidt) Glenn, C.W. Bacon & Hanlin) (Clavicipitaceae)] via the host grass *Festuca pratensis* Huds. (using the synonym *Lolium pratense* (Huds.) Darbysh.) (Poaceae) to the hemiparasite *Rhinantus serotinus* (Schönh.) Oborny (Orobanchaceae). The hemiparasite utilizes the alkaloids for protection against the herbivorous aphid *Aulacorthum solani* (Aphididae) [90]. Furthermore the transfer of glucosinolates from *Arabidopsis thaliana* (L.) Heynh. (Brassicaceae) to holoparasitic *Cuscuta gronovii* Willd. ex Roem. & Schult. (Convolvulaceae), the uptake of cardiac glycosides by hemiparasitic mistletoes [*Muellerina celastroides* (Schult. & Schult. f.) Tiegh., as *Phrygilanthus celastroides* (Schult. & Schult. f.) Eichler], *Dendrophthoe falcata* (L.f.) Ettingsh., and *Amyema congener* (Sieber ex Schult. & Schult.f.) Tiegh. (all Loranthaceae) from *Nerium oleander* L. (Apocynaceae), and the sequestration of cardenolides by holoparasitic *Cuscuta* species from *Digitalis lanata* Ehrh. and *Digitalis purpurea* L. (both Plantaginaceae) (after artificial infection) [91,92,93] were described as examples for plant-plant interactions.

### 2.5. Bioactivities Reported for Extracts Obtained from Orobanche Taxa and for Natural Products Isolated from Orobanche Taxa

Furthermore, biological activities of extracts or isolated pure compounds have been reported for some *Orobanche* species. Bioactivities of *Orobanche* species or of *Orobanche* natural products are compiled and listed alphabetically by activity in the following paragraphs.

#### 2.5.1. Analgesic Effects

Potent analgesic effects of the phenylpropanoid containing fraction of *O. crenata* extract (oral application) were observed in mice using the hot plate method for testing. *O. crenata* phenylpropanoid containing fraction was administered in doses of 50 mg/100 g body weight (b.w.) and 100 mg/100 g b.w. Paracetamol was used for comparison with a dose of 50 mg/100 g b.w. Reaction times were measured right after application and after 10, 20, 30, 45, 60, and 120 min after application. At the beginning of the experiment (0 min) the control group without any analgetic treatment, the *O. cernua* 50 mg/100 g b.w., *O. cernua* 100 mg/100 g b.w., and the paracetamol group showed reaction times of 16.1 ± 0.0, 17.8 ± 0.6, 14.3 ± 3.0, and 16.0 ± 1.3 s, respectively. Reaction times of the control group during the experiment varied slightly between 14.1 ± 1.1 s and 16.3 ± 0.1 s. Reaction times after application of 50 mg/100 g b.w. *O. cernua* extract increased from 17.8 ± 0.6 s at 0 min to reach their maximum of 22.6 ± 0.9 s after 20 min and decreased again afterwards. After application of 100 mg/100 g b.w. *O. cernua* extract, reaction times increased from 14.3 ± 3.0 s at 0 min to 33.7 ± 1.7 s after 45 min, and then decreased again. Paracetamol application (50 mg/100 g b.w.) led to an increase of the reaction time from 16.0 ± 1.3 s at 0 min to 28.4 ± 0.7 s after 45 min, followed by a decrease. The results implicate a clear analgesic effect of *O. cernua* phenylpropanoid containing fraction after oral application in mice [29]. Other studies on pharmacological effects of phenylpropanoids also revealed analgesic effects, especially of acteoside (**10**), one of the main phenylpropanoid glycosides found in *O*. species. Acteoside showed an analgesic potency almost equal to aminopyrine when tested in mice [94], and was effective against neuropathic pain in rats [95].

#### 2.5.2. Antimicrobial Activities

The phenolic composition of *O. crenata* 80% methanolic extract and its in vivo efficacy against fungal postharvest diseases were studied in an attempt to find new strategies for reducing postharvest diseases in sweet cherry fruit and replacing or integrating the use of synthetic fungicides. Sweet cherry fruit were sprayed with *O. crenata* extract (different concentrations: 1×, 2×, 4×; the 1× concentration corresponding to 0.170 mg dry matter/mL of buffer), *O. crenata* extract added with salts (CaCl_2_ or NaHCO_3_, 1% *w*/*v*), salt solutions, and the same buffer solution used to prepare the plant extracts (0.1 M K-phosphate, pH 5.5) as a positive control few hours after harvesting. Afterwards they were stored under controlled conditions. Rot incidence, expressed as the percentage of rotten fruit with respect to the total number of fruit in each tray, was assessed daily. At a rot incidence of around 50% the inhibition values of different treatments were evaluated. *O. crenata* extract inhibited postharvest rot in higher extract concentrations. An increase in extract concentration produced an increase in the percentage of inhibition from 64% to 76% for *O. crenata*. Addition of salt to the most concentrated extract further increased the inhibition of postharvest rot to 82% and 84% for NaHCO_3_ and CaCl_2_, respectively, and hereby proved to have a high antifungal efficacy [33]. Antifungal activity of *O. aegyptiaca* ethanolic and acetone extracts against *Fusarium oxysporum* Schlechtend., *Cladosporium harbarum* (Pers.) Link, *Trichothecium roseum* (Pers.) Link, and *Trichoderma viride* Pers. have been described by Nagaraja et al. [96]. Saadoun et al. tested *O. aegyptiaca*, *O. cernua*, and *O. crenata* ethanolic extracts against *Agrobacterium tumefaciens* (Smith & Townsend) Conn and *Erwinia* plant pathogens using the hole-plate diffusion method, the dilution method, and the Bauer–Kirby method. Tobromycin (10 μg), augmentin (30 μg), norfloxacin (10 μg), streptomycin (10 μg), ofloxacin (5 μg), cefuroxime (30 μg), and cefotaxime (30 μg) were tested as standard antibiotics for comparison. *O. cernua* showed inhibitory effects on *A. tumefaciens* isolates with MIC of 12,500 µg/mL, which is equal to streptomycin (10 μg), ofloxacin (5 mg) norfloxacin (10 μg), and cefotaxime (30 μg) activity against *Agrobacterium*. *Erwinia* isolates were less sensitive and higher concentrations of *Orobanche* extracts were needed for growth inhibition. *O. aegyptiaca* did not show antimicrobial activities in this study [97]. Nada et al. evaluated the antimicrobial activity of *O. crenata* extract against three gram positive bacteria (*Staphylococcus aureus* Rosenbach, *Bacillus subtilis* (Ehrenberg) Cohn, and *Streptococcus faecalis* (Andrewes & Horder) Schleifer & Kilpper-Bälz) and three gram negative bacteria *Escherichia coli* (Migula) Castellani & Chalmers, *Pseudomonas aeruginosa* Migula, and *Neisseria gonorrhoeae* (Zopf) Trevisan) using the disc diffusion assay. *O. crenata* showed moderate antibacterial activity against the investigated bacteria [35]. Abbes et al. tested antimicrobial activities of *O. crenata* and *O. foetida* methanolic and aqueous extracts using the disc diffusion method. Bacteria tested were *P. aeruginosa*, *E. coli*, *Enterococcus faecalis* (Andrewes & Horder) Schleifer & Kilpper-Bälz, *Enterobacter cloacae* (Jordan) Hormaeche & Edwards, *Salmonella enterica* (ex Kauffmann & Edwards) Le Minor & Popoff (subspecies: *Salmonella typhi* (*Salmonella enterica* subsp. *enterica* Serovar Typhi), *Salmonella enteritidis* (*Salmonella enterica* subsp. *enterica* Serovar Enteritidis), *Salmonella salamae* (*Salmonella enterica* subsp. *salamae*), *Shigella flexneri* Castellani & Chalmer, *S. aureus*, *Streptococcus pyogenes* Rosenbach, *Listeria monocytogenes* (Murray et al.) Pirie, *Yersinia enterocolitica* (Schleifstein & Coleman) Frederiksen, *Proteus mirabilis* Hauser, *Bacillus cereus* Frankland & Frankland, and *B. subtilis*. *O. crenata* methanolic extract was active against all tested bacteria except for *S. aureus, O. foetida* methanolic extract inhibited only *S. enteritidis* and *L. monocytogenes*. Aqueous extracts were not active againts the tested bacteria [37]. Antibacterial activity of an ethanolic extract of *O. cernua* at a concentration of 100 mg/mL in distilled water against five different bacteria species, four gram positive bacteria (*S. aureus*, *B. cereus*, *S. pyogenes*, *Streptococcus* sp.), and one gram negative bacterium (*E. coli*), was tested by Saadoun et al. For antimicrobial activity determination the hole-plate diffusion method, the dilution method, and the Bauer–Kirby method were applied. For evaluation of the minimum inhibitory concentration (MIC) the dilution method was used. Tobromycin (10 µg), nalidixic acid (30 µg), amoxicillin (30 µg), and cefotaxime (30 µg) were tested as standard antibiotics. *O. cernua* extract showed inhibitory activity against all tested bacteria with MIC of 1527, 3125, 25,000 and 50,000 µg/mL for *S. aureus*, *Streptococcus* sp., *S. pyogenes* and both for *B. cereus* and *E. coli,* respectively. In comparison with standard antibiotics an MIC of 3125 µg/mL is equal to cefotaxime (30 µg) and tobromycin (10 µg) activity against *Streptococcus* sp. and *S. aureus*, respectively; 25,000 and 50,000 µg/mL is equal to cefotaxime (30 µg) activity against *S. pyogenes* and *B. cereus*, respectively, and 50,000 µg/mL is equal to nalidixic acid (30 µg) activity against *E. coli* [98]. Antibacterial and antifungal activities in vitro of caffeic acid (**34**) and its derivatives including verbascoside (**10**) and orobanchoside (**29**) isolated from *O. rapum-genistae*, and poliumoside (**11**) (naturally occurring in *O. crenata* [14,36], *P. aegyptiaca* [56], and *P. ramosa* [14]) against two plant-pathogenic fungi (*Sclerotinia sclerotiorum* (Lib.) de Bary, *Botrytis cinerea* Pers. ex Nocca & Balb.) and seven plant-pathogenic bacteria (gram positive: *Corynebacterium rathayi* (Smith) Dowson, *Corynebacterium fascians* (Tilford) Dowson, *Corynebacterium sepedonicum* (Spieckermann & Kotthoff) Skaptason & Burkholder; gram negative: *A. tumefaciens*, *Erwinia carotovora* var. *carotovora* (Jones) Dye, *Xanthomonas pelargonii* (Brown) Starr & Burkholder, *Pseudomonas syringae* van Hall) were tested. The other tested substances were caffeic acid (**34**), ferulic acid (**36**), esculine, esculetin, rosmarinic acid, gallic acid methylester, chlorogenic acid (**37**), plantamajoside, and neomyricoside. Additionally *Forsythia intermedia* var. *spectablis* Koehne extract was tested. Ferulic acid (**36**) and chlorogenic acid (**37**) had been identified in *O. cernua* by other authors [17]. *E. coli* and *S. aureus* were used as references. For fungi MIC was evaluated in solid media using five different concentrations (0.12, 0.25, 0.50, 1.00, 2.00 mg/mL) of solutions of the tested substances, and for bacteria an agar diffusion method with four different concentrations of solutions of the tested substances (12.5, 25, 50, 100 mg/mL) together with MIC measurement in liquid media with six different concentrations of the tested substances (0.1, 0.5, 1.0, 1.5, 2.0, 2.5 mg/mL) were used. Orobanchoside (**29**) and caffeic acid (**34**) showed pronounced antifungal activities with MIC values of 2.00 mg/mL and 0.25 mg/mL, respectively, against *S. sclerotiorum* in media with pH 5. Orobanchoside (**29**) furthermore had an MIC value of 0.25 mg/mL against *S. sclerotiorum* and 1.00 mg/mL against *B. cinera* in media with pH 7, while caffeic acid (**34**) showed an MIC of 0.25 mg/mL against *B. cinera* in pH 7 medium. Verbascoside (**10**) and poliumoside (**11**) were both able to reduce growth of *S. sclerotiorum* and *B. cinera* in pH 5 and pH 7 media, but complete inhibition was not observed. Ferulic acid (**36**) showed MIC values of 0.13 mg/mL against *S. scleretorium* and *B. cinerea* in pH 5 and pH 7 media. Chlorogenic acid (**37**) was able to reduce growth of *S. sclerotiorum* and *B. cinerea* in pH 5 and pH 7 media, but again, complete inhibition was not observed. Against the tested bacteria MIC values were as follows: MIC of caffeic acid (**34**) against *C. rathayi* (1.0 mg/mL), *C. fascians* (1.0 mg/mL), *C. sepedonicum* (0.1 mg/mL), *A. tumefaciens* (1.0 mg/mL), *E.carotovora* var. *carotovora* (1.0 mg/mL), *X. pelargonii* (1.0 mg/mL), *P. syringae* (not determined), *S. aureus* (1.5 mg/mL), *E. coli* (1.5 mg/mL); MIC of ferulic acid (**36**) against *C. rathayi* (0.5 mg/mL), *C. fascians* (0.5 mg/mL), *C. sepedonicum* (0.5 mg/mL), *A. tumefaciens* (1.0 mg/mL), *E.carotovora* var. *carotovora* (1.0 mg/mL), *X. pelargonii* (0.5 mg/mL), *P. syringae* (1.0 mg/mL), *S. aureus* (1.0 mg/mL), *E. coli* (1.5 mg/mL); MIC of chlorogenic acid (**37**) against *C. rathayi* (1.5 mg/mL), *C. fascians* (1.0 mg/mL), *C. sepedonicum* (1.0 mg/mL), *A. tumefaciens* (2.0 mg/mL), *E.carotovora* var. *carotovora* (2.5 mg/mL), *X. pelargonii* (1.5 mg/mL), *P. syringae* (2.0 mg/mL), *S. aureus* (>2.5 mg/mL), *E. coli* (2.5 mg/mL); MIC values of verbascoside (**10**), poliumoside (**11**), and orobanchoside (**29**) were not investigated. Caffeic acid (**34**) and its derivatives are potential natural plant protective agents against some plant-pathogenic fungi and bacteria as demonstrated in this work. Streptomycin, tested along with the caffeic acid derivatives, was a much more potent bacterial growth inhibitor than the other tested compounds with MIC values of <0.1 mg/mL *(C. rathayi, C. fascians, C. sepedonicum, A. tumefaciens, E.carotovora* var. *carotovora, S. aureus, E. coli*), with an exception for *X. pelargonii* (>2.5 mg/mL) (*P. syringae* MIC not determined) [99].

#### 2.5.3. Antioxidant Activities as Food Preservative

*O. crenata* ethanolic extract total antioxidant activity was tested using the phosphomolybdenum method with ascorbic acid as standard. The antioxidant activity was expressed as ascorbic acid equivalents (AE) (mg/g of extract). The two investigated individual *Orobanche* plants showed good total antioxidant activity 619 ± 9 mg AE/g extract and 561 ± 9 mg AE/g extract [35].

#### 2.5.4. Antioxidative Effects, Anti-Inflammatory Activity in Human Leucocytes, Effects on Production of Reactive Oxygen Species (ROS)

Phenylpropanoid glycosides isolated from *O. coerulescens* were tested for their antioxidative effects on human low-density lipoprotein. For evaluation of their antioxidant activity dialyzed LDL obtained from human blood samples was diluted with PBS to 100 µg/mL, pre-incubated with the test compounds at 37 °C for 30 min, and then incubated with CuSO_4_ at 37 °C to induce lipid peroxidation. Resveratrol, a natural phenolic antioxidant e.g., from red wine, was used as a positive control. Conjugated diene formation was monitored and prolonged lag phase (min) used as an index of antioxidant activity when an antioxidant was present in LDL oxidation with Cu^2+^. All seven isolated phytochemical compounds, phenylpropanoid glycosides desrhamnosyl acteoside (**9**), acteoside (**10**), caerulescenoside (**13**), campneoside II (**17**), isoacteoside (**23**), oraposide (**29**), and 3′-methyl crenatoside (**30**) suppressed conjugated diene formation with IC_50_ values of 0.64 ± 0.03, 0.31 ± 0.01, 1.25 ± 0.06, 1.15 ± 0.04, 1.01 ± 0.05, 1.69 ± 0.15, and 2.97 ± 0.31 µM, respectively while resveratrol had an IC_50_ value of 6.75 ± 1.05 µM. This showed that all isolated compounds from *O. coerulescens* were more effective antioxidants than the positive control, resveratrol [23]. Phenylpropanoid glycosides acteoside (**10**), rossicaside B (**28**), and oraposide (**29**), isolated from *O. coerulescens*, were tested for their inflammation-modulating activity in human leucocytes. Peripheral human neutrophils (PMNs) and mononuclear cells were exposed to phorbol-12-myristate-13-acetate (PMA), a direct proetin kinase C (PKC) activator, and *N*-formyl-methionyl-leucocyl-phenylalanine (fMLP), a receptor mediated and G protein coupled activator, for the induction of production of reactive oxygen species (ROS) and upregulation of β2 integrin in an in-vitro model. For the prevention of PMA-induced ROS production, acteoside (**10**), rossicaside B (**28**), and oraposide (**29**) showed IC_50_ values of 12.8 ± 7.2 µM, 5.6 ± 2.8 µM, and 6.8 ± 2.3 µM respectively in PMNs, and IC_50_ values of 9.6 ± 3.2 µM, 23.9 ± 2.9 µM, and 10.0 ± 4.3 µM respectively in mononuclear cells. IC_50_ values for prevention of fMLP-induced ROS production were 3.5 ± 0.6 µM, 3.0 ± 0.1 µM, and 3.0 ± 0.2 µM for acteoside (**10**), rossicaside B (**28**), and oraposide (**29**) in PMNs, respectively, and 8.8 ± 3.2 µM, 3.5 ± 0.2 µM, and 3.5 ± 0.2 µM in mononuclear cells, respectively. Furthermore, the inhibition of NADPH oxidase (NOX) activity in cell lysate by phenylpropanoid glycosides was tested since NOX is the major ROS producing enzyme in activated leucocytes. Acteoside (**10**), rossicaside B (**28**), and oraposide (**29**) were more potent in NOX inhibition than the positive control, diphenyleneiodonium (DPI, a NOX inhibitor). Moreover, these compounds showed effective free radical-scavenging activity in a cell-free DPPH (2,2-diphenyl-1-picrylhydrazyl) assaying system. Acteoside (**10**) and oraposide (**29**) also significantly inhibited PMA- and fMLP-induced β2 integrin expression in human peripheral leucocytes. These effects make *O. coerulescens* and other drugs containing acteoside (**10**), rossicaside B (**28**), and oraposide (**29**) interesting as potential anti-inflammatory agents for the treatment of oxidative-stress-related diseases [24]. Antioxidative potential was also investigated by Kidachi et al. for phenylpropanoids acteoside (**10**), cistanoside D (**20**), isoacteoside (**23**), oraposide (**29**), 3‴-*O*-methyl crenatoside (**30**), and isocrenatoside (**32**) from a methanolic *O. minor* extract, two synthetic derivatives acteoside-tetramethylether, oraposide-tetramethylether, as well as caffeic acid (**34**) and hydroxytyrosol using the DPPH (2,2-diphenyl-1-picrylhydrazyl) radical scavenging activity assay. Strong activities were observed for acteoside (**10**) (IC_50_ 15.2 µM), isoacteoside (**23**) (IC_50_ 20.0 µM), oraposide (**29**) (IC_50_ 24.5 µM), and isocrenatoside (**32**) (IC_50_ 29.0 µM), whereas moderate activities were observed for 3‴-*O*-methyl crenatoside (**30**) (IC_50_ 54.2 µM), caffeic acid (**34**) (IC_50_ 38.7 µM), and hydroxytyrosol (IC_50_ 44.6 µM), and no antioxidant activities were observed for acteoside-tetramethylether (IC_50_ > 100 μM), oraposide-tetramethylether (IC_50_ > 100 μM), and cistanoside D (**20**) (IC_50_ > 100 μM). Epigallocatechin gallate used as positive control showed an IC_50_ value of 13.5 µM. No standard deviations of the measured values were indicated [46]. Gao et al. found *O. cernua* extract and acteoside (**10**) to exhibit strong scavenging effects with IC_50_ values of 56.3 µg/mL and 20.6 µg/mL, respectively. No standard deviations of the measured values were given [100]. Antioxidant activities of *O. crenata* and *O. foetida* methanolic and aqueous extracts was tested by Abbes et al. using DPPH and ABTS (2,2-azino-bis-3-ethylbenzothiazoline-6-sulfonic acid) radical scavenging activity assays. Synthetic antioxidants BHT (2,6-di-tert-butyl-4-methylphenol) and AA (ascorbic acid) were used as positive controls. At 1.00 µg/mL DPPH radical scavenging activities of *O. crenata* methanolic extract, *O.crenata* water extract, *O. foetida* methanolic extract, *O. foetida* water extract, BHT, and AA were 19.5, 18.3, 5.86, 14.7, 13.4, and 54.0%, respectively. At 200 µg/mL *O. crenata* methanolic extract, *O.crenata* water extract, *O. foetida* methanolic extract, *O. foetida* water extract, BHT, and AA showed DPPH radical scavenging activities of 88.1, 77.0, 92.0 86.1, 85.0, and 86.3%, respectively. The highest activity against DPPH radicals was observed for *O. foetida* methanolic extract with an IC_50_ value of 7.19 ± 1.75 µg/mL (BHT: IC_50_ 65.5 ± 1.4 µg/mL; AA IC_50_ 0.93 ± 0.07 µg/mL). Antioxidant activities in the ABTS test, expressed in % inhibition at 0.5 µg/mL, were 4.04 (*O. crenata* methanolic extract), 1.64 (*O. crenata* aqueous extract), 1.28 (*O. foetida* methanolic extract), and 2.34 (*O. foetida* aqueous extract) (BHT: 9.98%; AA: 23.1%). Activities of nearly 100% for *O. crenata* methanolic extract, *O.crenata* water extract, *O. foetida* methanolic extract, *O. foetida* water extract, BHT, and AA, respectively, were observed at concentrations of 200 µg/mL [37].

#### 2.5.5. Blood Pressure and Blood Platelet Aggregation

Intravenous injection of the glycosidic fraction of *O. crenata* 70% ethanolic extract into rats in doses up to 20 mg/100 g led to a temporary lowering of the arterial blood pressure of the treated animals. Higher doses caused slight, persistent lowering of the arterial blood pressure [29]. Hypotensive activity of *O. aegyptiaca* 30% aqueous extract and of the alkaloid containing chloroform fraction (further fractionation of the extract with different solvents gave hexane, ether, chloroform, alcohol, and water fractions) after i.v. injection into dogs was also evaluated. The alkaloidal fraction showed strong hypotensive effects. (Hypertension was artificially induced using the Goldblatt technique.) 10 mg i.v. lowered the blood pressure by about 48 mmHg for three hours [57]. A mixture of verbascoside (**10**) and orobanchoside (**29**) extracted from *O. hederae* was tested for its effect on ADP-induced (10–15 µM) blood platelet aggregation and blood pressure in New Zealand male rabbits and Wistar male rats. A dose-dependent inhibition of ADP-induced platelet aggregation of 12.9 ± 4.0%, 43.7 ± 7.8%, 49.4 ± 6.4%, 59.4 ± 6.9%, and 73.7 ± 8.3% at concentrations of 0.2 mg/mL, 0.4 mg/mL, 0.6 mg/mL, 0.8 mg/mL, and 1.0 mg/mL phenylpropanoid glycosides respectively was observed using an aggregometer. Blood pressure was not affected by phenylpropanoid glycosides injected i.v. into the test animals [42].

#### 2.5.6. Contractions of Toad and Rabbit Hearts and Rat Intestines

*O. aegyptiaca* 30% aqueous extract was further fractionated with different solvents to give hexane, ether, chloroform, alcohol, and water fractions. The extract and fractions were tested for different biological activities. Effects on toad (*Bufo regularis* Reuss) and rabbit hearts were investigated. Doses of 1, 2, 3, and 4 mL of the aqueous extract were added to 50 mL bath (Ringer´s solution for toad hearts, Lock´s solution for rabbit hearts) and the amplitude or heart rate (toad hearts) as well as the volume of Lock´s solution perfused by the heart (rabbit hearts) were recorded. Contractions of toad hearts and rabbits’ hearts perfused by the extract were stimulated. Also contractions of isolated rats intestines were stimulated whereas uterine contractions in rats were inhibited [57].

#### 2.5.7. Diuretic Effects

Oral application of the phenylpropanoid containing fraction of *O. crenata* extract in rats had strong diuretic effects. *O. crenata* extract doses of 100 mg/100 g body weight and 200 mg/100 g body weight were orally applied. Rats were put in diuresis cages and the volume of the collected urine was measured after 1, 3, 6, and 24 h. The untreated control group produced 0, 2.65 ± 0.22, 6.25 ± 0.05, and 11.2 ± 0.1 mL urine after 1, 3, 6, and 24 h, respectively. After application of *O. crenata* extract doses of 100 mg/100 g b.w. 0, 3.27 ± 0.05, 6.95 ± 0.05, and 12.9 ± 0.1 mL urine were collected after 1, 3, 6, and 24 h, respectively. *O. crenata* extract doses of 200 mg/100 g b.w. led to 0.37 ± 0.05, 3.95 ± 0.05, 8.25 ± 0.12, and 14.3 ± 0.2 mL of urine after 1, 3, 6, and 24 h, respectively, showing increasing diuresis with higher *O. cernua* extract doses [29]. *O. aegyptiaca* 30% aqueous extract was further fractionated with different solvents to give hexane, ether, chloroform, alcohol, and water fractions. The extract and fractions were tested for different biological activities. Diuretic effects of the 20% alcoholic extract were observed in rabbits. Urine volumes of treated animals were measured after 0.5, 1, 2, 3, and 24 h and compared to urine volumes of the animals after 24 h without any treatment. The average urine volume of treated animals (average dose of 9.5 mL of 20% extract/kg b.w.) after 24 h was 107 ± 51 in comparison with 73 ± 21.2 mL for the untreated animals [57].

#### 2.5.8. Inhibition of Amyloid β-Aggregation

Acteoside (**10**) and oraposide (**29**) isolated from *O. minor* were tested for their inhibitory effects on aggregation of human 42-mer amyloid β-protein (Aβ-42), which is believed to play an important role in the pathogenesis of Alzheimer´s disease. Thioflavin-T (Th-T) fluorescence assays, transmission electron microscopy (TEM), and circular dichroism (CD) spectroscopy were used to investigate the inhibitory effects. Acteoside (**10**) and oraposide (**29**) showed potent inhibitory effects on the aggregation of Aβ-42 with IC_50_ values of 8.9 µM and 3.6 µM respectively. IC_50_ values were calculated from the inhibition rate (%) of each compound towards Aβ-42 aggregation after 24 h by using the Th-T assay. Furthermore, an anti-aggregating effect was suggested by the significant reduction of Aβ fibril formation by 50 µM acteoside (**10**). β-Sheet formation in Aβ-42 was also inhibited [45]. Kidachi et al. also tested inhibition of amyloid β-42 (Aβ-42) aggregation by phenylpropanoids acteoside (**10**) and oraposide (**29**) from *O. minor* methanolic extract, their synthetic derivatives acteoside-tetramethylether, oraposide-tetramethylether, as well as cistanoside D (**20**), isoacteoside (**23**), 3‴-*O*-methyl crenatoside (**30**), isocrenatoside (**32**), caffeic acid (**34**), and hydroxytyrosol. The IC_50_ values were calculated from the inhibitory rate (%) of each compound towards Aβ-42 aggregation after 48 h by using the thioflavin-T (Th-T) fluorescence assay. Aβ-42 aggregation was inhibited by acteoside (**10**) and oraposide (**29**) with IC_50_ values of 11.3 µM and 8.2 µM, respectively. Moderate inhibitory activity was observed for 3‴-*O*-methyl crenatoside (**30**) (IC_50_ 28.0 µM), very weak inhibitory activity was observed for caffeic acid (**34**) (IC_50_ 93.8 μM) and hydroxytyrosol (IC_50_ 92.0 μM), and no inhibitory activity was observed for acteoside-tetramethylether (IC_50_ > 100 μM), oraposide-tetramethylether (IC_50_ > 100 μM), and cistanoside D (**20**) (IC_50_ > 100 μM). 3,4-Di-*O*-caffeoylquinic acid used as positive control for Aβ-42 aggregation showed an IC_50_ value of 30.2 µM. No standard deviations of the measured values were indicated [46]. The observed anti-amyloidal effects make acteoside (**10**) a potential agent for treating or preventing Alzheimer’s disease [45].

#### 2.5.9. Memory Enhancing Effects

Acteoside (**10**) showed memory enhancing effects and increased significantly the expression of nerve growth factor (NGF) and tropomycin receptor kinase A (TrkA) mRNA and protein in the hippocampus in mice [13]. NGF and TrkA are closely associated with cognitive function and a decrease thereof is related to Alzheimer´s disease. Acteoside (**10**) treatment resulted in an improvement of learning and memory deficits via promotion of NGF and TrkA expression in the brain. The authors used a senescent mouse model induced by a combination of chronic intraperitoneal administration of D-gal (60 mg/kg/day) and oral administration of AlCl_3_ (5 mg/kg/day) once daily for 90 days. After 60 days mice in three different groups were treated intragastrically with acteoside (**10**) (30, 60, and 120 mg/kg/day) for 30 days. Learning ability and memory of the mice were tested using the Morris water maze test. Afterwards mice brains were removed and the hippocampus CA1 region studied immunohistochemically. Reverse transcription polymerase chain reactions (RT-PCR) and western blot analyses were performed to investigate the expression of NGF mRNA and TrkA mRNA [13].

#### 2.5.10. Muscle Relaxant and Antispasmodic Effects

Dose dependent smooth muscle relaxant effects (phenylpropanoid containing fraction of *O. crenata* extract) were observed when testing different doses on the peristaltic movements of isolated perfused rabbit’s intestine. Doses of 50, 80, 100, 150, and 200 mg/50 mL bath were tested. Movement inhibitions of 31.9 ± 6.8, 38.4 ± 10.1, 44.2 ± 4.3, 51.2 ± 10.3, and 95.4 ± 2.7% were obsereved, respectively [29]. Potent antispasmodic effects on isolated perfused guinea-pig ileum were observed (phenylpropanoid fraction of *O. crenata* extract). Contractions were induced by acetylcholine application and afterwards different doses of *O. crenata* extract were tested for their antispasmodic potential. Extract doses tested were 200, 400, 600, and 800 mg/50 mL bath. Inhibition of spasmodic contractions ranged from 22.8 ± 2.1, 55.6 ± 3.7, and 67.3 ± 10.1 to 94.3 ± 6.4%, respectively, showing a dose dependent antispasmodic potential [29].

#### 2.5.11. Nutrient Source

*O. crenata* was found to be a good source of nutrients. It contained a low moisture level (<8%), a high amount of protein (7.30%), ash contents of 9.20–10.1%, a crude fiber content ranging from 22.1 to 23.5%, and a nutritive value of 244–247 kcal/100 g plant dry weight [35].

#### 2.5.12. Photoprotective Effects

*O. cernua* ethanolic extract and its principal component, acteoside (**10**), were studied for their photoprotective effects on UVB-induced photoaging as well as for the underlying molecular mechanisms in normal human dermal fibroblasts (NHDFs). UV radiation causes excessive reactive oxygen species (ROS) generation, which triggers matrix metalloproteinase (MMPs) production, collagen degradation, and premature aging (photoaging). Cell viability of UVB-irradiated NHDFs and the effects of *O. cernua* extract and acteoside (**10**) on cell viability were tested using the 3-(4,5-dimethylthiazol-2-yl)-2,5-diphenyltetrazolium bromide (MTT) assay. *O. cernua* extract (100 µg/mL) and acteoside (**10**) (10 µM) were able to recover cell viability by 24.7% and 26.9%, respectively. For effects of *O. cernua* extract and acteoside (**10**) on intracellular ROS generation cells were first exposed to UVB irradiation, which increased the ROS level by 282% (ROS levels of normal group were set to 100%). The following treatment with *O. cernua* extract (100 µg/mL) and acteoside (**10**) (10 µM) reduced ROS levels by 73.0% and 42.3%, respectively. Furthermore, *O. cernua* extract and acteoside (**10**) significantly reduced MMP-1 and IL-6 (Interleukin) levels in NHDFs exposed to UVB-irradiation. *O. cernua* extract (100 µg/mL) and acteoside (**10**) (10 µM)-treated groups suppressed UVB-induced MMP-1 levels by 49.0% and 57.1%, respectively. Additionally, the secretion of IL-6 was lowered by 79.4% and 57.1%, by *O. cernua* extract (100 µg/mL) and acteoside (**10**) (10 µM). *O. cernua* extract and acteoside (**10**) could reverse a UVB induced decrease in type-I procollagen mRNA, with an increased rate of 52.7% and 25.7%, respectively. Furthermore, the UVB-induced increased production of MMP-1 and MMP-3 mRNA levels were strongly inhibited by *O. cernua* extract (100 µg/mL) and acteoside (**10**) (10 µM). *O. cernua* extract decreased the expression of MMP-1 and MMP-3 mRNA by 42.5% and 28.3%, respectively, while acteoside (**10**) decreased the expression by 44.4% and 66.7%, respectively. Carried with the AP-1 binding sites, the promoters of MMPs were transactivated by AP-1 transcription factor. The expression of phosphorylated c-fos and c-jun, the major components of AP-1 was measured and inhibitory effects on UVB-induced p-c-fos and p-c-jun expression in a dose-dependent manner by acteoside (**10**) treatments were observed. *O. cernua* extract (100 µg/mL) reduced the levels of p-c-fos and p-c-jun by 56.0% and 75.6%, respectively, and acteoside (**10**) (10 µM) reduced the levels by 93.0% and 65.3%, respectively. The mitigen-activated protein kinase (MAPK) signaling pathway, as the upstream of AP-1 transcription factor, has been reported to be activated by UVB-elevated ROS. Biological effects of *O. cernua* extract and acteoside (**10**) on the MAPKs family were further studied in UVB-irradiated NHDFs. UVB radiation elevated the phosphorylated forms of MAPKs molecules including ERK, JNK and p38. *O. cernua* extract and acteoside (**10**) suppressed the phosphorylation of ERK, JNK and p38 caused by UVB. Levels of p-ERK, p-JNK and p-38 were decreased by *O. cernua* (100 µg/mL) extract by 46.4%, 58.8% and 84.8%, respectively, and decreased by acteoside (**10**) (10 µM) treatment by 47.9%, 75.5%, and 77.4%, respectively. The effects of *O. cernua* extract and acteoside (**10**) on Nrf2 nuclear translocation and antioxidant enzyme expression were investigated in Western blots in UVB-irradiated NHDFs. Data showed that the nuclear levels already raised by UVB-irradiation were further elevated by *O. cernua* extract and acteoside (**10**). The expression of Nrf2 was increased by 56.0% and 69.0% by *O. cernua* extract (100 µg/mL) and acteoside (**10**) (10 µM), respectively. Moreover, HO-1 and NQO-1 levels were increased by *O. cernua* extract (100 µg/mL) by 76.4% and 120%, and by acteoside (**10**) (10 µM) by 103% and 110%, respectively. Furthermore, *O. cernua* extract and acteoside (**10**) were able to reverse the downregulation of TGF-β1 and *p*-Smad2/3 expression in UVB-irradiated NHDFs. TGF-β1and p-Smad2/3 protein expressions were recovered by 71.6% and 70.7% by *O. cernua* extract (100 µg/mL), and by 53.7% and 182%, respectively, by acteoside (**10**) (10 µM), compared with the UVB radiation group. Also *O. cernua* extract (100 µg/mL) and acteoside (**10**) (10 µM) inhibited the UVB-induced Smad7 expression by 48.9% and 57.1%, respectively, in comparison with the UVC group. The antiphotoaging effects of *O. cernua* extract and acteoside (**10**) were investigated and it was detected that *O. cernua* extract and acteoside (**10**) inhibited UVB-irradiated MMP-1 and MMP-3 mRNA upregulation and IL-6 secretion. Moreover, *O. cernua* extract and acteoside (**10**) reduced UVB-induced MMP-1 protein secretion, and enhanced type-I procollagen synthesis in NHDFs. *O. cernua* extract and acteoside (**10**) treatment furthermore led to the inhibition of the UVB-activated MAPK/AP-1 pathway by inhibiting the UVB-induced phosphorylation of ERK, JNK, and p38 and the expression of p-c-fos and p-c-jun. Levels of cytoprotective agents HO-1 and NQO-1 were increased by *O. cernua* extract and acteoside, hereby increasing protection against UVB-induced oxidative stress through activation of the cutaneous endogenous antioxidant system. UVB-induced enhacement of Smad7 expression and decrease of Smad2/phosphorylation were reversed by *O. cernua* extract and acteoside (**10**), and TGF-β1 expression was enhanced, hereby repairing the TGF-β/Smad signaling pathway and enhancing type-I procollagen synthesis [100]. (No standard deviations of the measured values described above were indicated.)

#### 2.5.13. Summary of Bioactivities

In conclusion, *Orobanche* extracts and isolated *Orobanche* natural products were positively tested for a variety of biological activities including anti-hypertensive, anti-platelet aggregating, and memory enhancing effects. UV protecting and anti-photoaging effects open an interesting field of study and antioxidant activities on human LDL, inflammation modulating effects in human leucocytes, ROS production and amyloid β-aggregation inhibiting effects make the species containing the responsible substances potential agents for treatment of Alzheimer’s disease and oxidation related diseases. Furthermore, *Orobanche* extracts are active against a wide variety of pathogenic fungi and bacteria and can be potential alternatives to synthetic antibiotics and plant protecting agents. The by far best investigated compound is the phenylpropanoid glycoside acteoside (**10**) which is responsible for a large part of the observed effects, such as antioxidant, anti-inflammatory, radical scavenging, amyloid β-aggregation inhibiting, memory enhancing, antimicrobial, and photoprotective effects and also oraposide (**29**) was shown to have several interesting effects. However, the occurrence of acteoside (**10**) is not restricted to *Orobanche* or Orobanchaceae but the compound is widely distributed in the plant kingdom. It is found in over 200 species belonging to 23 plant families, most of them belonging to the order Lamiales [101]. Thus, even though *Orobanche* extracts and substances extracted thereof show the above stated biological activities, there might be better and easier accessible sources for the bioactive compounds than the holoparasitic taxa of the genus *Orobanche*.

## 3. Discussion

Most of the natural products found in the genus *Orobanche* (*n* = 70) have so far been reported only from one source (*n* = 51), and only three compounds from more than four taxa: acteoside **10** (from 13 source taxa), oraposide **29** (from 12 source taxa), and orobanone **45** (also from 12 source taxa). While most of the literature on *Orobanche* is about strigolactones, seed germination stimulants, parasitic weed management, and host-parasite interaction (SciFinder, last accessed first of October, 2018), publications on secondary metabolism of *Orobanche* species are relatively rare. Of the more than 200 species belonging to *Orobanche* s.l. only 27 species have been investigated for secondary metabolites. Compound classes detected in the analyzed species comprise aromatic aldehydes, ketones and phenylmethanoids (Figure 1), phenylethanoids (Figure 2), phenylethanoid glycosides (Figure 3), phenylpropanoid glycosides (Figure 4, Figure 5, Figure 6, Figure 7 and Figure 8), phenolic acids (Figure 9), lignans (Figure 10 and Figure 11), flavonoids (Figure 12 and Figure 13), a tropone derivative (Figure 14), and sterols (Figure 15, Figure 16, Figure 17, Figure 18, Figure 19 and Figure 20). Investigations on biological activities of *Orobanche* extracts and isolated pure secondary metabolites from *Orobanche* species show a wide variety of effects, e.g., antibacterial and antifungal activities [35,46,98], inhibition of amyloid-β-aggregation [45,46] or photoprotection against UVB-irradiation [100]. *Orobanche* are not only destructive weeds, but might also be a source of active agents against several diseases, in particular against fungal and bacterial, and inflammatory diseases, correlated with ROS production. Nevertheless, it has to be considered, that phenylpropanoids in general and e.g., acteoside, one of the best investigated compounds of *Orobanche* in particular, are not restricted to *Orobanche* species but are widely distributed in the plant kingdom, possibly making other species more interesting sources of these compounds [101,102]. An aspect that deserves more research and could be a challenging subject for future studies is the idea that natural products sequestered by *Orobanche* species from their host species could be further metabolized by the parasites. Metabolization of host plant natural products could result in new, formerly undescribed hybrid compounds not synthesized by a single species. To study this phenomenon, more analytical studies of the secondary metabolism of *Orobanche* species and their host plants are warranted.

## Figures and Tables

**Table:** 

No	Name	R_1_	R_2_	R_3_
**1**	*p*-Hydroxybenzaldehyde	H	OH	H
**2**	Protocatechuic aldehyde	OH	OH	H
**3**	Isovanillin	OH	OCH_3_	H
**4**	Vanillin	OCH_3_	OH	H
**5**	Syringaldehyde	OCH_3_	OH	OCH_3_

**Table:** 

No	Name	R_1_	R_2_
**7**	2-Phenylethyl β-primeveroside	H	Xyl
**8**	Salidroside ^1^	OH	H

^1^ Syn. rhodioloside.

**Table:** 

No	Name	R_1_	R_2_	R_3_	R_4_	R_5_	R_6_
**9**	Desrhamnosyl acteoside	H	H	H	H	H	H
**10**	Acteoside ^1^	H	H	H	H	Rha	H
**11**	Poliumoside	H	H	H	H	Rha	Rha
**12**	Arenarioside	H	H	H	H	Rha	Xyl
**13**	Caerulescenoside	H	H	H	H	(3-*O*-Glc)-Rha	H
**14**	2′-*O*-Acetylacteoside	H	H	H	Ac	Rha	H
**15**	2′-*O*-Acetylpoliumoside ^2^	H	H	H	Ac	Rha	Rha
**16**	Pheliposide	H	H	H	Ac	Rha	Xyl
**17**	Campneoside II	H	H	OH	H	Rha	H
**18**	Campneoside I	H	H	OCH_3_	H	Rha	H
**19**	Leucosceptoside A	CH_3_	H	H	H	Rha	H
**20**	Cistanoside D	CH_3_	CH_3_	H	H	Rha	H
**21**	Orobancheoside B	CH_3_	CH_3_	H	H	Rha	feruloyl-
**22**	Orobancheoside A	CH_3_	CH_3_	H	H	(4-*O*-Ac)-Rha	H

^1^ Syn. kusaginin [16], orobanchin, verbascoside; ^2^ syn. brandioside.

**Table:** 

No	Name	R
**23**	Isoacteoside ^1^	H
**24**	Isocampneoside I ^2^	OCH_3_

^1^ Syn. isoverbascoside, ^2^ stereochemistry not reported.

**Table:** 

No	Name	R
**29**	Oraposide ^1^	H
**30**	3‴-*O*-Methyl crenatoside	CH_3_

^1^ Syn. crenatoside, orobanchoside.

**Table:** 

No	Name	R
**31**	Descaffeoyl crenatoside	H
**32**	Isocrenatoside	caffeoyl-
**33**	3′-*O*-Methyl isocrenatoside	ferulouyl-

**Table:** 

No	Name	R_1_	R_2_
**34**	Caffeic acid	H	H
**35**	Methyl caffeate	H	CH_3_
**36**	*trans*-Ferulic acid	CH_3_	H

**Table:** 

No	Name	R_1_	R_2_
**39**	(+)-Pinoresinol 4′-*O*-β-D-glucopyranoside	H	H
**40**	Isoeucommin A	H	OCH_3_
**41**	(+)-Syringaresinol 4′-*O*-β-D-glucopyranoside	OCH_3_	OCH_3_

**Table:** 

No	Name	R_1_	R_2_
**42**	Apigenin	H	H
**43**	Luteolin	OH	H
**44**	Tricin	OCH_3_	OCH_3_

**Table:** 

No	Name	R_1_	R_2_
**47**	Cholesterol	H	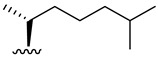
**48**	Campesterol	H	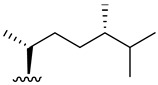
**49**	24-Methylene cholesterol	H	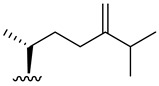
**50**	β-Sitosterol	H	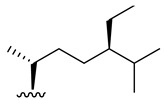
**51**	Stigmasterol	H	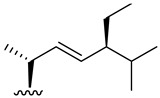
**52**	*Iso*fucosterol	H	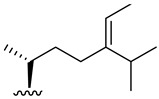
**53**	Daucosterol	β-D-Glc	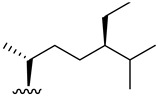
**54**	Stigmasterol-3-*O*-β-D-glucoside	β-D-Glc	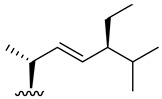

**Table:** 

No	Name	R_1_	R_2_
**55**	∆^7^-Campestenol	H	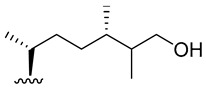
**56**	Episterol	OH	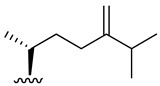
**57**	∆^7^-Stigmastenol	OH	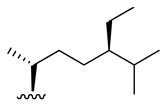
**58**	∆^7^-Avenasterol	OH	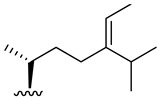

**Table:** 

No	Name	R
**59**	24-Methyl lophenol	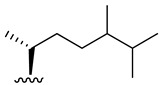
**60**	24-Methylene lophenol	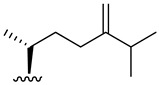
**61**	24-Ethyl lophenol	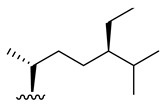
**62**	24-Ethylidene lophenol	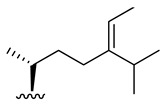

**Table:** 

No	Name	R_1_	R_2_
**63**	4α-Methyl ∆^8^-campestenol	H	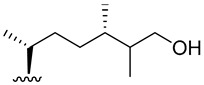
**64**	4α-Methyl ∆^8^-stigmastenol	OH	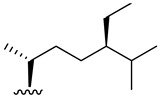
**65**	24,28-Dihydro obtusifoliol	OH	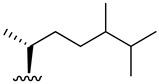
**66**	Obtusifoliol	OH	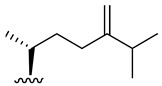

**Table:** 

No	Name	R_1_	R_2_
**67**	24,28-Dihydrocycloeucalenol	H	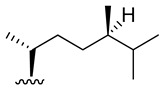
**68**	Cycloeucalenol	H	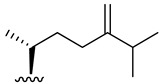
**69**	Cycloartenol	CH_3_	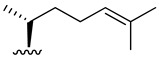
**70**	24-Methylene cycloartenol	CH_3_	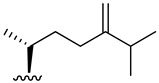

**Table:** 

No	Name	R_1_	R_2_	R_3_	R_4_
**S2**	(−)-Sparteine	H, H	H	H	H, H
**S3**	(−)-17-Oxosparteine	H, H	H	H	=O
**S4**	(+)-Lupanine	=O	H	H	H, H
**S5**	17-Oxolupanine	=O	H	H	=O
**S6**	(+)-13-Hydroxylupanine	=O	H	OH	H, H
**S7**	13-Tigloyloxylupanine ^1^	=O	H	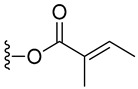	H, H
**S8**	13-Angeloyloxylupanine ^1^	=O	H	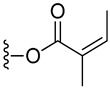	H, H
**S9**	13-Benzoyloxylupanine ^1^	=O	H	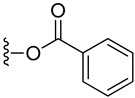	H, H
**S10**	4-Hydroxylupanine	=O	OH	H	H, H

^1^ Stereochemistry not resolved.

## Supplementary Materials

The following are available online. Table S1: Overview Natural Products synthesized by *Orobanche* species. Table S2: Natural Products sequestered by *Orobanche* species from host species. Text S1: Literature search strategy & key words.

## References

[1] Kojić M., Maširević S., Jovanović D. (2001). Distribution and biodiversity of broomrape (*Orobanche* L.) worldwide and in Serbia. Helia.

[2] Pusch J., Günther K.-F., Wagenitz G. (2009). Orobanchaceae. Gustav Hegi—Illustrierte Flora von Mitteleuropa, Band VI Teil 1A, Dez. 2009//Orobanchaceae (Sommerwurzgewächse), Scrophulariaceae (Rachenblütler).

[3] Park J.-M., Manen J.-F., Colwell A.E., Schneeweiss G.M. (2008). A plastid gene phylogeny of the non-photosynthetic parasitic *Orobanche* (Orobanchaceae) and related genera. J. Plant Res..

[4] Schneeweiss G.M., Joel D.M., Gressel J., Musselman L.J. (2013). Phylogenetic relationships and evolutionary trends in Orobanchaceae. Parasitic Orobanchaceae—Parasitic Mechanisms and Control Strategies.

[5] Schneider A.C. (2016). Resurrection of the genus Aphyllon for New World broomrapes (*Orobanche* s.l., Orobanchaceae). PhytoKeys.

[6] Rubiales D., Westwood J., Uludag A. (2009). Proceedings IPPS International Parasitic Plant Society 10th World Congress of Parasitic Plants. http://parasiticplants.org/docs/IPPS_10th_Congress_Abstracts_Kusadasi_Turkey.pdf.

[7] Joel D.M. (2009). The new nomenclature of *Orobanche* and *Phelipanche*. Weed Res..

[8] Bennett J.R., Mathews S. (2006). Phylogeny of the parasitic plant family Orobanchaceae inferred from phytochrome A. Am. J. Bot..

[9] Schneeweiss G.M., Colwell A., Park J.-M., Jang C.-G., Stuessy T.F. (2004). Phylogeny of holoparasitic *Orobanche* (Orobanchaceae) inferred from nuclear ITS sequences. Mol. Phylogenet. Evol..

[10] The Plant List, a Working List of All Plant Species Scientific Plant Names of Vascular Plants and Bryophytes. Collaboration between the Royal Botanic Gardens, Kew and Missouri Botanical Garden. http://www.theplantlist.org.

[11] Roudbaraki S.J., Nori-Shargh D. (2016). The volatile constituent analysis of *Orobanche alba* Stephan from Iran. Curr. Anal. Chem..

[12] Fruchier A., Rascol J.-P., Andary C., Privat G. (1981). A tropone derivative from *Orobanche rapum-genistae*. Phytochemistry.

[13] Gao L., Peng X.-M., Huo S.-X., Liu X.-M., Yan M. (2015). Memory enhancement of acteoside (verbascoside) in a senescent mice model induced by a combination of D-gal and AlCl_3_. Phytother. Res..

[14] Serafini M., Di Fabio A., Foddai S., Ballero M., Poli F. (1995). The occurrence of phenylpropanoid glycosides in Italian *Orobanche* spp.. Biochem. Syst. Ecol..

[15] Aynehchi Y., Salehi Sormaghi M.H., Amin G.H., Soltani A., Qumehr N. (1982). Survey of Iranian plants for saponins, alkaloids, flavonoids and tannins. II. Int. J. Crude Drug Res..

[16] Mølgaard P., Ravn H. (1988). Evolutionary aspects of caffeoyl ester distribution in dicotyledons. Phytochemistry.

[17] Qu Z.-Y., Zhang Y.-W., Yao C.-L., Jin Y.-P., Zheng P.-H., Sun C.-H., Liu J.-X., Wang Y.-S., Wang Y.-P. (2015). Chemical constituents from *Orobanche cernua* Loefling. Biochem. Syst. Ecol..

[18] Qu Z.-Y., Zhang Y.-W., Zheng S.-W., Yao C.-L., Jin Y.-P., Zheng P.-H., Sun C.-H., Wang Y.-P. (2016). A new phenylethanoid glycoside from *Orobanche cernua* Loefling. Nat. Prod. Res..

[19] Yang M.-Z., Wang X.-Q., Li C. (2014). Chemical constituents from *Orobanche cernua*. Zhongcaoyao.

[20] Zhao J., Liu T., Ma L., Yan M., Zhao Y., Gu Z., Huang Y. (2009). Protective effect of acteoside on immunological liver injury induced by *Bacillus Calmette-Guerin* plus lipopolysaccharide. Planta Med..

[21] Zhao J., Yan M., Huang Y., Liu T., Zhao Y. (2009). Study on water soluble constituents of *Orobanche coerulescens*. Tianran Chanwu Yanjiu Yu Kaifa.

[22] Murayama T., Yanagisawa Y., Kasahara A., Onodera K.-I., Kurimoto M., Ikeda M. (1998). A novel phenylethanoid, isocrenatoside isolated from the whole plant of *Orobanche coerulescens*. J. Nat. Med..

[23] Lin L.-C., Chiou W.-F., Chou C.-J. (2004). Phenylpropanoid glycosides from *Orobanche caerulescens*. Planta Med..

[24] Lin L.-C., Wang Y.-H., Hou Y.-C., Chang S., Liou K.-T., Chou Y.-C., Wang W.-Y., Shen Y.-C. (2006). The inhibitory effect of phenylpropanoid glycosides and iridoid glucosides on free radical production and beta2 integrin expression in human leucocytes. J. Pharm. Pharmacol..

[25] Wang L.-J., Yang Q., Wang F., Zhu P. (2016). A new phenethyl alcohol glycoside from *Orobanche coerulescens*. Zhongcaoyao.

[26] Zhao J., Yan M., Huang Y., He W.-Y., Zhao Y. (2007). Study on chemical constituents of *Orobanche coerulescens*. Zhongyaocai.

[27] Shao H.-X., Yang J.-Y., Ju A.-H. (2011). Studies on chemical constituents of Mongolian medicine *Orobanche coerulescens*. Zhonghua Zhongyiyao Zazhi.

[28] Zhang Q.-R. (2017). A new phenethyl alcohol glycoside from *Orobanche coerulescens*. Zhongguo Zhong Yao Za Zhi.

[29] El-Shabrawy O.A., Melek F.R., Ibrahim M., Radwan A.S. (1989). Pharmacological evaluation of the glycosidated phenylpropanoids containing fraction from *Orobanche crenata*. Arch. Pharm. Res..

[30] Afifi M.S., Lahloub M.F., El-Khayaat S.A., Anklin C.G., Rüegger H., Sticher O. (1993). Crenatoside: A novel phenylpropanoid glycoside from *Orobanche crenata*. Planta Med..

[31] Gatto M.A., Sanzani S.M., Tardia P., Linsalata V., Pieralice M., Sergio L., Di Venere D. (2013). Antifungal activity of total and fractionated phenolic extracts from two wild edible herbs. Nat. Sci..

[32] Gatto M.A., Ippolito A., Linsalata V., Cascarano N.A., Nigro F., Vanadia S., Di Venere D. (2011). Activity of extracts from wild edible herbs against postharvest fungal diseases of fruit and vegetables. Postharvest Biol. Technol..

[33] Gatto M.A., Sergio L., Ippolito A., Di Venere D. (2016). Phenolic extracts from wild edible plants to control postharvest diseases of sweet cherry fruit. Postharvest Biol. Technol..

[34] Nishibe S., Tamayama Y., Sasahara M., Andary C. (1995). A phenylethanoid glycoside from *Plantago asiatica*. Phytochemistry.

[35] Nada S.A., El-Chaghaby G.A. (2015). Nutritional evaluation, phytoconstituents analysis and biological activity of the parasitic plant *Orobanche crenata*. J. Chem. Biol. Sci. Sect. A.

[36] Dini I., Iodice C., Ramundo E. (1995). Phenolic metabolites from *Orobanche speciosa*. Planta Med..

[37] Abbes Z., El Abed N., Amri M., Kharrat M., Ben Hadj Ahmed S. (2014). Antioxidant and antibacterial activities of the parasitic plants *Orobanche foetida* and *Orobanche crenata* on faba bean in Tunisia. J. Anim. Plant Sci..

[38] Andary C., Wylde R., Maury L., Heitz A., Dubourg A., Nishibe S. (1994). X-ray analysis and extended NMR study of oraposide. Phytochemistry.

[39] Andary C., Wylde R., Laffite C., Privat G., Winternitz F. (1982). Structures of verbascoside and orobanchoside, caffeic acid sugar esters from *Orobanche rapum-genistae*. Phytochemistry.

[40] Aynilian G.H., Abou-Char C.I., Edgecombe W. (1971). Screening of herbarium specimens of native plants from the families Amaranthaceae, Dipsaceae and Orobanchaceae for alkaloids, saponins and tannins. Planta Med..

[41] Pieretti S., Di Giannuario A., Capasso A., Nicoletti M. (1992). Pharmacological effects of phenylpropanoid glycosides from *Orobanche hederae*. Phytother. Res..

[42] Capasso A., Pieretti S., Di Giannuario A., Nicoletti M. (1993). Pharmacological study of phenylpropanoid glycosides: Platelet aggregation and blood pressure studies in rabbits and rats. Phytother. Res..

[43] Baccarini A., Melandri B.A. (1967). Studies on *Orobanche hederae* physiology: Pigments and CO_2_ fixation. Physiol. Plant..

[44] Rohmer M., Ourisson G., Benveniste P., Bimpson T. (1975). Sterol biosynthesis in heterotrophic plant parasites. Phytochemistry.

[45] Kurisu M., Miyamae Y., Murakami K., Han J., Isoda H., Irie K., Shigemori H. (2013). Inhibition of amyloid β aggregation by acteoside, a phenylethanoid glycoside. Biosci. Biotechnol. Biochem..

[46] Kidachi E., Kurisu M., Miyamae Y., Hanaki M., Murakami K., Irie K., Shigemori H. (2016). Structure-activity relationship of phenylethanoid glycosides on the inhibition of amyloid β aggregation. Heterocycles.

[47] Dzhumyrko S.F., Sergeeva N.V. (1985). Carotenoid pigments from *Orobanche owerinii*. Chem. Nat. Compd..

[48] Han J.-X., Yang J.-Y., Shao H.-X., Ju A.-H. (2010). A study on the chemical constituents of *Orobanche pycnostachya* Hance. Neimenggu Daxue Xuebao Ziran Kexueban.

[49] Li C.-F., Wen A.-P., Wang X.-Q., Han G.-Q. (2016). HPLC simultaneous determination of three phenylethanoid glycosides in *Orobanche pycnostachya*. Yaowu Fenxi Zazhi.

[50] Andary C., Privat G., Chevallet P., Orzalesi H., Serrano J.J., Boucard M. (1980). Chemical and pharmacodynamic study of heteroside esters of caffeic acid, isolated from *Orobanche rapum-genistae*. Farm. Sci..

[51] Andary C., Rascol J.P., Privat G. (1980). Two new ecotypes of *Orobanche rapum-genistae* Thuill. Trav. Soc. Pharm. Montp..

[52] Bridel M., Charaux C. (1924). L’orobanchine, glucoside nouveau, retiré des tubercules de l’*Orobanche rapum* Thuill. Bull. Soc. Chim. Fr..

[53] Bridel M., Charaux C. (1925). Sur le processus du noircissement des *Orobanches* au cours de leur dessiccation. C. R. Hebd. Seanc. Acad. Sci..

[54] Viron C., Lhermite S., André P., Lafosse M. (1998). Isolation of phenylpropanoid glycosides from *Orobanche rapum* by high speed countercurrent chromatography. Phytochem. Anal..

[55] Viron C., Pennanec R., André P., Lafosse M. (2000). Large scale centrifugal partition chromatography in purification of polyphenols from *Orobanche rapum*. J. Liq. Chrom..

[56] Afifi M.S.A., Lahloub M.F., Zaghloul A.M., El-Khayaat S.A. (1993). Phenylpropanoid glycosides from *Orobanche aegyptiaca* and *Orobanche ramosa*. J. Pharm. Sci..

[57] Sharaf A., Youssef M. (1971). Pharmacologic investigation on *Orobanche egyptiaca* with a special study on its hypotensive action. Qual. Plant. Mater. Veg..

[58] Lahloub M.F., Zaghloul A.M., El-Khayaat S.A., Afifi M.S., Sticher O. (1991). 2′-*O*-Acetylpoliumoside: A new phenylpropanoid glycoside from *Orobanche ramosa*. Planta Med..

[59] Harborne J.B. (1958). Identification of the flavone pigment of *Phelipaea ramosa*. Chem. Ind..

[60] Melek F.R., Aboutabl E.A., Elsehrawy H. (1992). Tricin from *Orobanche ramosa* L.. Egypt. J. Pharm. Sci..

[61] Andary C., Privat G., Wylde R., Heitz A. (1985). Pheliposide et arenarioside, deux nouveaux esters hétérosidiques de l’acide caféique isolés de *Orobanche arenaria*. J. Nat. Prod..

[62] Smith J.D., Mescher M.C., de Moraes C.M. (2013). Implications of bioactive solute transfer from hosts to parasitic plants. Curr. Opin. Plant Biol..

[63] Lotti G., Paradossi C. (1977). Host-parasite mineral composition in plants infected with Orobanchaceae. Agric. Ital..

[64] Lotti G., Paradossi C. (1987). Absorption of petroselinic acid by *Orobanche hederae* on *Hedera helix*. Agrochimica.

[65] Sareedenchai V., Zidorn C. (2008). Sequestration of polyacetylenes by the parasite *Orobanche hederae* (Orobanchaceae) from its host *Hedera helix* (Araliaceae). Biochem. Syst. Ecol..

[66] Wink M., Witte L., Hartmann T. (1981). Quinolizidine alkaloid composition of plants and of photomixotrophic cell suspension cultures of *Sarothamnus scoparius* and *Orobanche rapum-genistae*. Planta Med..

[67] Rascol J.P., Andary C., Roussel J.L., Privat G. (1978). Alkaloids from *Orobanche rapum-genistae*. I. Isolation and identification of three major alkaloids, (−)-sparteine, (+)-lupanine, and (+)-13-hydroxylupanine. Plantes Med. Phytother..

[68] Rascol J.P., Andary C., Privat G. (1980). Alkaloids of *Orobanche rapum-genistae*. II. Variation of the amount of sparteine and lupanine in the host-parasite relations. Trav. Soc. Pharm. Montp..

[69] Fournier G., Paris M. (1983). Mise en évidence de cannabinoïds chez *Phelipaea ramosa*, Orobanchacées, parasitant le chanvre, *Cannabis sativa*, Cannabinacées. Planta Med..

[70] Mebs D. (2001). Toxicity in animals. Trends in evolution?. Toxicon.

[71] Bornancin L., Bonnard I., Mills S.C., Banaigs B. (2017). Chemical mediation as a structuring element in marine gastropod predator-prey interactions. Nat. Prod. Rep..

[72] Erb M., Robert C.A.M. (2016). Sequestration of plant secondary metabolites by insect herbivores: Molecular mechanisms and ecological consequences. Curr. Opin. Insect Sci..

[73] Nishida R. (2002). Sequestration of defensive substances from plants by *Lepidoptera*. Annu. Rev. Entomol..

[74] Opitz S.E.W., Müller C. (2009). Plant chemistry and insect sequestration. Chemoecology.

[75] Harborne J.B. (2001). Twenty-five years of chemical ecology. Nat. Prod. Rep..

[76] Rasmussen L.S., Rank C., Jensen S.R. (2006). Transfer of iridoid glucosides from host plant *Galium verum* to hemiparasitic *Euphrasia stricta*. Biochem. Syst. Ecol..

[77] Stermitz F.R., Foderaro T.A., Li Y.-X. (1993). Iridoid glycoside uptake by *Castilleja integra* via root parasitism on *Penstemon teucrioides*. Phytochemistry.

[78] Stermitz F.R., Harris G.H. (1987). Transfer of pyrrolizidine and quinolizidine alkaloids to *Castilleja* (Scrophulariaceae) hemiparasites from composite and legume host plants. J. Chem. Ecol..

[79] Arslanian R.L., Harris G.H., Stermitz F.R. (1990). New quinolizidine alkaloids from *Lupinus argenteus* and its hosted root parasite *Castilleja sulphurea*. Stereochemistry and conformation of some naturally occurring cyclic carbinolamides. J. Org. Chem..

[80] Adler L.S., Wink M. (2001). Transfer of quinolizidine alkaloids from hosts to hemiparasites in two *Castilleja-Lupinus* associations: Analysis of floral and vegetative tissues. Biochem. Syst. Ecol..

[81] Boros C.A., Marshall D.R., Caterino C.R., Stermitz F.R. (1991). Iridoid and phenylpropanoid glycosides from *Orthocarpus* spp. Alkaloid content as a consequence of parasitism on *Lupinus*. J. Nat. Prod..

[82] Wink M., Witte L. (1993). Quinolizidine alkaloids in *Genista acanthoclada* and its holoparasite, *Cuscuta palaestina*. J. Chem. Ecol..

[83] Bäumel P., Witte L., Czygan F.-C., Proksch P. (1994). Transfer of qinolizidine alkaloids from various host plants of the Fabaceae to parasitizing *Cuscuta* species. Biochem. Syst. Ecol..

[84] Schneider M.J., Stermitz F.R. (1990). Uptake of host plant alkaloids by root parasitic *Pedicularis* species. Phytochemistry.

[85] Cabezas N.J., Urzúa A.M., Niemeyer H.M. (2009). Translocation of isoquinoline alkaloids to the hemiparasite, *Tristerix verticillatus* from its host, *Berberis montana*. Biochem. Syst. Ecol..

[86] Marko M.D., Stermitz F.R. (1997). Transfer of alkaloids from *Delphinium* to *Castilleja* via root parasitism. Norditerpenoid alkaloid analysis by electrospray mass spectrometry. Biochem. Syst. Ecol..

[87] Martín-Cordero C., Ayuso M.A., Richomme P., Bruneton J. (1989). Quinolizidine alkaloids from *Viscum cruciatum*, hemiparasitic shrub of *Lygos sphaerocarpa*. Planta Med..

[88] Cordero C.M., Serrano A.G., Gonzalez M.A. (1993). Transfer of bipiperidyl and quinolizidine alkaloids to *Viscum cruciatum* Sieber (Loranthaceae) hemiparasitic on *Retama sphaerocarpa* Boissier (Leguminosae). J. Chem. Ecol..

[89] Martín-Cordero C., Pedraza M.A., Gil A.M., Ayuso M.J. (1997). Bipiperidyl and quinolizidine alkaloids in fruits of *Viscum cruciatum* hemiparasitic on *Retama sphaerocarpa*. J. Chem. Ecol..

[90] Lehtonen P., Helander M., Wink M., Sporer F., Saikkonen K. (2005). Transfer of endophyte-origin defensive alkaloids from a grass to a hemiparasitic plant. Ecol. Lett..

[91] Smith J.D., Woldemariam M.G., Mescher M.C., Jander G., de Moraes C.M. (2016). Glucosinolates from host plantsinfluence growth of the parasitic plant *Cuscuta gronovii* and its susceptibility to aphid feeding. Plant Physiol..

[92] Boonsong C., Wright S.E. (1961). The cardiac glycosides present in mistletoes growing on *Nerium oleander*. Aust. J. Chem..

[93] Rothe K., Diettrich B., Rahfeld B., Luckner M. (1999). Uptake of phloem-specific cardenolides by *Cuscuta* sp. growing on *Digitalis lanata* and *Digitalis purpurea*. Phytochemistry.

[94] Nakamura T., Okuyama E., Tsukada A., Yamazaki M., Satake M., Nishibe S., Deyama T., Moriya A., Maruno M., Nishimura H. (1997). Acteoside as the analgesic principle of cedron (*Lippia triphylla*), a Peruvian medicinal plant. Chem. Pharm. Bull..

[95] Isacchi B., Iacopi R., Bergonzi M.C., Ghelardini C., Galeotti N., Norcini M., Vivoli E., Vincieri F.F., Bilia A.R. (2011). Antihyperalgesic activity of verbascoside in two models of neuropathic pain. J. Pharm. Pharmacol..

[96] Nagaraja T.G., Nare R.B., Laxmikant V., Patil B. (2010). In vitro screening of antimicrobial activity of *Orobanche aegyptiaca*. J. Biopestic..

[97] Saadoun I., Hameed K.M., Al-Momani F., Ababneh Q. (2008). Effect of three *Orobanche* spp. extracts on some local phytopathogens, *Agrobacterium* and *Erwinia*. Turk. J. Biol..

[98] Saadoun I., Hameed K.M. (1999). Antibacterial activity of *Orobanche cernua* extract. J. Basic Microbiol..

[99] Ravn H., Andary C., Kovács G., Mølgaard P. (1989). Caffeic acid esters as in vitro inhibitors of plant pathogenic bacteria and fungi. Biochem. Syst. Ecol..

[100] Gao W., Wang Y.-S., Qu Z.-Y., Hwang E., Ngo H.T.T., Wang Y.-P., Bae J., Yi T.-H. (2018). *Orobanche cernua* Loefling attenuates ultraviolet B-mediated photoaging in human dermal fibroblasts. Photochem. Photobiol..

[101] Alipieva K., Korkina L., Orhan I.E., Georgiev M.I. (2014). Verbascoside—A review of its occurrence, (bio)synthesis and pharmacological significance. Biotechnol. Adv..

[102] Kurkin V.A. (2003). Phenylpropanoids from medicinal plants: Distribution, classification, structural analysis, and biological activity. Chem. Nat. Compd..

