# Supplementary material for: Genuine and Sequestered Natural Products from the Genus Orobanche (Orobanchaceae, Lamiales)
Source: Molecules. 2018 Oct 30;23(11):2821. doi: 10.3390/molecules23112821 (PMC6278508; doi:10.3390/molecules23112821)
Supplement: Supplementary file 1 [file molecules-23-02821-s001.zip › Supplementary_Text_1.pdf]

Scharenberg & Zidorn: *Orobanche* - Supplementary Text 1

Literature search strategy & keywords

For literature search on natural products synthesized by *Orobanche* species as well as their bioactivities the following search terms were used: [*Orobanche*], [*Orobanche* AND activity], [*Orobanche* AND anthocyanins], [*Orobanche* AND bioactivity], [*Orobanche* AND chemical AND compounds], [*Orobanche* AND chemistry], [*Orobanche* AND components], [*Orobanche* AND compounds], [*Orobanche* AND constituents], [*Orobanche* AND flavonoids], [*Orobanche* AND iridoids], [*Orobanche* AND metabolites], [*Orobanche* AND phenethyl AND alcohol AND glycoside], [*Orobanche* AND phenylethanoids], [*Orobanche* AND phenylpropanoids], [*Orobanche* AND phenylpropanoid AND glycosides], [*Orobanche* AND phytochemistry], and [*Orobanche* AND secondary AND metabolites]. Literature data on classification of Orobanchaceae was done based on the search for [Orobanchaceae], [Orobanchaceae AND systematics], [Orobanchaceae AND chemosystematics], [Orobanchaceae AND phytochemistry], [Orobanchaceae AND phylogeny], [Orobanchaceae AND classification], and [Orobanchaceae AND taxonomy], and reports of sequestration of natural products by parasitic plant and animal species were retrieved using the keywords [*Orobanche* AND sequestration], [Herbivores AND sequestration], [hemiparasites AND sequestration], [insects AND sequestration], [plants AND sequestration], [plants AND interactions].
